# DHX8 regulates degradation of RNA by RNautophagy

**DOI:** 10.1093/nar/gkaf801

**Published:** 2025-08-21

**Authors:** Ryohei Sakai, Eigo Takeda, Chihana Kabuta, Viorica Raluca Contu, Yuuki Fujiwara, Nobuhiro Fujikake, Tadafumi Hashimoto, Yoshinori Ohsumi, Tomohiro Kabuta

**Affiliations:** Department of Degenerative Neurological Diseases, National Institute of Neuroscience, National Center of Neurology and Psychiatry, Tokyo 187-8502, Japan; Cell Biology Center, Institute of Innovative Research, Tokyo Institute of Technology, Yokohama 226-8503, Japan; Department of Degenerative Neurological Diseases, National Institute of Neuroscience, National Center of Neurology and Psychiatry, Tokyo 187-8502, Japan; Department of Degenerative Neurological Diseases, National Institute of Neuroscience, National Center of Neurology and Psychiatry, Tokyo 187-8502, Japan; Department of Degenerative Neurological Diseases, National Institute of Neuroscience, National Center of Neurology and Psychiatry, Tokyo 187-8502, Japan; Department of Degenerative Neurological Diseases, National Institute of Neuroscience, National Center of Neurology and Psychiatry, Tokyo 187-8502, Japan; Department of Degenerative Neurological Diseases, National Institute of Neuroscience, National Center of Neurology and Psychiatry, Tokyo 187-8502, Japan; Cell Biology Center, Institute of Innovative Research, Tokyo Institute of Technology, Yokohama 226-8503, Japan; Department of Degenerative Neurological Diseases, National Institute of Neuroscience, National Center of Neurology and Psychiatry, Tokyo 187-8502, Japan

## Abstract

RNautophagy is an intracellular degradation pathway in which RNA is directly taken up by lysosomes. The cytoplasmic regions of the lysosomal membrane proteins, LAMP2C and SIDT2, can interact with consecutive guanine sequences in RNA, mediating the uptake of RNA during RNautophagy. RNautophagy has also been implicated in the clearance of expanded CAG-repeat mRNA and RNA foci associated with polyQ disease. However, the mechanisms of RNA uptake during RNautophagy remain unclear. Here, we screened for proteins that bind consecutive guanine sequences and identified RNA helicase DHX8 as a binding partner. DHX8 interacts with SIDT2 and is partially localized to the cytoplasmic side of the lysosomal membrane. We found that DHX8 regulates intracellular RNA degradation via SIDT2-dependent RNautophagy but not via macroautophagy. RNA binding, but not ATPase activity, of DHX8 is likely to be important for regulating RNA degradation. DHX8 also contributes to the clearance of pathogenic CAG repeat mRNA and RNA foci, and the levels of both soluble protein and insoluble high-molecular-weight aggregates of expanded polyQ tracts. Our findings provide insights into the mechanisms underlying the regulation of intracellular RNA degradation, autophagic pathways, and possibly the pathogenesis of repeat RNA-related disorders.

## Graphical Abstract

**Graphical Abstract:**
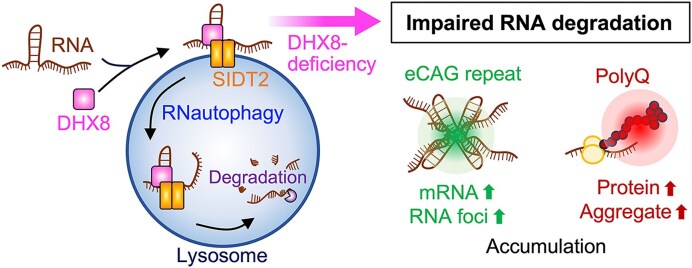

## Introduction

Synthesis and degradation are essential for maintaining biological homeostasis. The dysfunction of RNA-degrading enzymes can lead to diseases, demonstrating the crucial role of RNA degradation. For example, loss-of-function mutations in *RNASET2*, which is critical for RNA degradation via lysosomes, cause leucoencephalopathy, characterized by white matter abnormalities and neurological deficits [1–3]. Mutations in *EXOSC3*, a component of the exosome complex, a multiprotein complex essential for RNA degradation and processing in the cytoplasm or nucleus, lead to pontocerebellar hypoplasia characterized by cerebellar and brainstem underdevelopment, ataxia, and developmental delays [4].

RNautophagy and DNautophagy (RDA) are intracellular degradation pathways in which RNA and DNA, respectively, are directly taken up by lysosomes through mechanisms distinct from those of conventional macroautophagy [5–11]. Our previous studies have shown that the lysosomal membrane proteins LAMP2C and SIDT2 mediate nucleic acid uptake during RDA [5–10]. SIDT2 knockout (KO) mice exhibit accumulation of ribosomal RNA (rRNA), messenger RNA (mRNA), and proteins in muscle tissue, leading to rimmed vacuolar myopathy, highlighting the critical role of SIDT2-dependent degradation in muscle homeostasis [12]. Additionally, the importance of RNautophagy is underscored by its role in degrading pathogenic expanded CAG repeat (eCAGr)-containing RNAs implicated in neurodegenerative diseases, such as Huntington’s disease [9, 13].

RNA molecules undergo conformational changes via RNA helicases. RNA helicases modulate the secondary structure of RNA, thereby influencing RNA–protein interactions and controlling its intracellular functionality, stability, and degradation [14, 15]. Nevertheless, the mechanistic relationship between RNautophagy and RNA helicases remains to be elucidated [5].

Both SIDT2 and LAMP2C bind to nucleic acids through distinct arginine-rich motifs in the cytoplasmic regions [9, 16]. Among oligonucleotides composed of a single nucleotide base (poly-A, poly-C, poly-G, poly-U, poly-dA, poly-dC, poly-dG, and poly-dT), SIDT2 and LAMP2C bind specifically to poly-G and poly-dG [7, 9]. In addition, *in vitro* assays using isolated lysosomes demonstrated that among these oligonucleotides, lysosomes only take up poly-G and poly-dG [7].

Thus, we hypothesized that other RNautophagy-related factors also bind to consecutive guanine sequences and investigated the involvement of RNA helicases in RNautophagy by screening for proteins that bind to consecutive guanine nucleotides.

## Materials and methods

### Oligonucleotides

Poly-dA (15-mer): [Biotin]- 5′-AAAAAAAAAAAAAAA-3′; Poly-dG (15-mer): [Biotin]- 5′-GGGGGGGGGGGGGGG-3′. These biotin-labeled oligonucleotides were synthesized by FASMAC Co. Alexa568-labeled poly-dG (15-mer) was synthesized by JBioS.

### Plasmids

The pCI-neo-3 × HA-biotin (HB) construct was created by subcloning synthetic DNA encoding HA_3_-Biotin (purchased from IDT) into a pCI-neo mammalian expression vector (Promega, Cat#E1841). pCI-neo-hDHX8 and pCI-neo-hDHX8-HB were constructed by subcloning the complementary DNA (cDNA) encoding human DHX8 (RIKEN BRC DNA Bank, Cat#HGY042724) into pCI-neo and pCI-neo-HB, respectively. pCI-neo-hDHX8-R620A, pCI-neo-hDHX8-R620A-HB, pCI-neo-hDHX8-NT-HB, pCI-neo-hDHX8-CT-HB, pCI-neo-hDHX8-RecA-HB, and pCI-neo-hDHX8-WRO-HB were generated by introducing a mutation into pCI-neo-hDHX8 and pCI-neo-hDHX8-HB, respectively, using the KOD-Plus-Mutagenesis Kit (TOYOBO, Cat#SMK-101). pEGFP-N1-LAMP1, pTagBFP-N-LAMP1, and pmCherry-N1-SIDT2 were constructed by subcloning cDNA encoding human LAMP1 or SIDT2 into pEGFP-N1 (Clontech, product number unavailable), pTagBFP-N (Evrogen, Cat#FP172), and pmCherry-N1 (Clontech, Cat#632523), respectively. All the resulting plasmid constructs were analyzed using sequencing. pCI-neo-mSIDT2, pEGFP-N1-mSIDT2 [8], pTRE-Tight-HTTex1-CAG-22-EGFP, pTRE-Tight-HTTex1-CAG-145-EGFP [9], and pCI-neo-FLAG-mSIDT2 [10] plasmids were prepared as previously described. The pHR-tdMCP-YFP (Addgene, Cat#99151), pHR-Tre3G-47 × CAG-12 × MS2 (Addgene, Cat#99148), pTet-Off Advanced Vector (TAKARA, Cat#631070), and pTet-Off Vector (Clontech, Cat#631017) were purchased.

### Biotin-poly-dG or dA pull-down assay

Brain lysates were prepared from the 16-week-old C57BL/6J Jcl. (CLEA Japan) by lysing the tissue with 1% Triton lysis buffer [50 mM Tris–HCl (pH 7.4), 150 mM NaCl, 5 mM EDTA (pH 8.0), and 1% Triton X-100]. The lysate concentration was adjusted to 3 mg/ml. For the pull-down assay, 3 mg (1 ml) of the brain lysate was mixed with 1 nmol (10 μl) of biotin-poly-dG or biotin-poly-dA was added, followed by the addition of 30 μl of Streptavidin Sepharose High Performance beads (GE Healthcare, Cat#17-5113-01). The mixture was then incubated by rotating the tubes at 4°C overnight. After incubation, the beads were washed four times. For elution, 100 μl of 1× sample buffer (10 mM Tris, pH 7.8, 3% sodium dodecyl sulfate (SDS), 5% glycerol, 0.02% bromophenol blue, and 2% 2-mercaptoethanol) was added to the beads, and the mixture was incubated to elute the bound components. The resulting eluate was subjected to silver staining using a 2D-silver stain II kit (Daiichi, Cat#423413), immunoblot analysis, and mass spectrometry. All animal procedures were approved by the Animal Ethics Committee of the National Center of Neurology and Psychiatry.

### Analysis of ternary complex

N2a cells were transfected with pCI-neo-FLAG-mSIDT2 alone, pCI-neo-DHX8 alone, or both pCI-neo-FLAG-mSIDT2 and pCI-neo-DHX8. The pull-down assay was performed using biotin-poly-dG, following the same procedure as described in the “Biotin-poly-dG or dA pull-down assay” section.

### Peptide precipitation and LC-MS/MS

Each sample volume was adjusted to 200 μl by adding ultrapure water. Methanol (800 μl; Wako, Cat#138-14 521), chloroform (200 μl; Kishida Chemical, Co., Ltd., Cat#282-16 011), and 800 μl ultrapure water were added to the samples, with vortexing after each addition. The samples were centrifuged at 15 000 × *g* for 2 min, and the upper layer was discarded. Then, 600 μl of methanol was added, samples centrifuged at 15 000 × *g* for 2 min, and the resulting supernatants were discarded. Pellets were dried and dissolved in 50 μl of phase transfer surfactant solution [0.1 M Tris–HCl pH 9.0, 12 mM sodium deoxycholate (Wako, Cat#192-08 312), 12 mM sodium *N*-lauroyl sarcosinate (Wako, Cat#192–10 382)]. 0.5 μl of 1 M dithiothreitol (Nacalai Tesque, Cat#14112-52) in 50 mM NH_4_HCO_3_ (Wako, Cat#018-21742) was added to samples before samples were incubated at 42°C for 30 min. Next, 2.5 μl of 50 mM iodoacetamide (Wako, Cat#095-02151) in 50 mM NH_4_HCO_3_ was added to the samples, which were incubated at 25°C for 30 min in the dark. After adding 200 μl of 50 mM NH_4_HCO_3_, each sample was treated with 0.5 μg Lys-C (Wako, Cat#125-05061) for 3 h and followed by 0.5 μg trypsin gold (Promega, Cat#V5280) for 18 h at 37°C. 250 μl of ethyl acetate (Wako, Cat#051-00356) and a final concentration of 0.5% trifluoroacetic acid (Wako, Cat#208-02741) were added to the samples. The samples were vortexed for 2 min and centrifuged at 15 700 × *g* for 2 min. The upper layers were discarded and the lower layers were dried using a centrifugal evaporator (TAITEC, VC-96R, 0062458-000). Finally, peptides were purified using C18-Stagetip (CDS, Empore^™^ Discs, C18). Purified peptides were dried and dissolved in 15 μl of 2% acetonitrile (Wako, Cat#012-19851) and 0.1% formic acid (Kanto Chemical., Co., Inc., Cat#16233-96). Liquid chromatography was performed using an EASY-nLC 1000 (Thermo Fisher Scientific) equipped with a 125 mm × 75 μm C18 separation column (Nikkyo Technos, Co., Ltd.), and mass spectrometry was performed using a Q-Exactive mass spectrometer (Thermo Fisher Scientific). Separations were performed in the presence of 0.1% formic acid on a 0%–30% acetonitrile gradient for 60 min, followed by a 30%–100% acetonitrile gradient for 2 min and a further 8 min in the presence of 100% acetonitrile. Data were acquired in data-dependent acquisition mode using Xcalibur 4.0 software (Thermo Fisher Scientific). The settings of the data-dependent acquisition were as follows: the resolution was 70 000 for full MS and 17 500 for MS^2^, the AGC target was 1.0E6 for full MS and 5.0E5 for MS^2^, the maximum injection time was 60 ms for both full MS and MS^2^, the scan range was 300–2 000 *m/z* for full MS, the top 10 signals were selected for MS^2^, and the isolation window was 3.0 *m/z* for MS^2^. Data were acquired thrice for each sample. Acquired data were analyzed using Proteome Discoverer 2.4 (Thermo Fisher Scientific) to obtain abundance ratios and *P*-values between samples, and are shown with abundances (MS^1^-based quantification, total of three times) (Supplementary Table S1). Fragmentation spectra were searched against the *Mus musculus* UniProt database (version Nov. 19, 2019) containing 17 020 entries. The settings of Proteome Discoverer were as follows: maximum missed cleavages, 2; peptide length, 5–144; peptide mass tolerance, 10 ppm; fragment mass tolerance, ±0.02 Da. Meanwhile, oxidation, +15.995 Da (Met); propionamide, +71.037 Da (Cys); phospho, +79.966 Da (Ser, Thr, Tyr); GG dipeptide, +114.043 Da (Lys); Acetyl, +42.011 Da (N-terminus); Met-loss, –131.040 Da (N-terminus); and Met loss + Acetyl, –89.030 Da (N-terminus) were configured as variable modifications. Peptides were filtered to a 1% false discovery rate (FDR) target.

### Cell culture

*Atg5* ^–/–^ KO and *Atg13* KO mouse embryonic fibroblasts (MEFs) kind gifts from Dr. Noboru Mizushima (The University of Tokyo, Tokyo, Japan) [17, 18]. *Atg5* KO, *Atg13* KO, *Sidt2*^–/–^ (KO), and *Sidt2*^+/+^ (wild-type, WT) MEFs [12] and the mouse neuroblastoma cell line Neuro2a (N2a) cells were grown in Dulbecco’s modified Eagle’s medium (Gibco, Cat#C11995500CP) supplemented with 10% fetal bovine serum (for MEFs: NICHIREI, Cat#175 012, for N2a: SIGMA, Cat#F7524) at 37°C under humidified 5% CO_2_ atmosphere. Cells were passaged at 80%–100% confluency.

### Generation of stable cell lines

A lentiviral vector (pLV[Exp]-Bsd-CMV > ATXN3Q79) expressing ATXN3-Q79 was generated by VectorBuilder based on Addgene plasmid #22129 (pFLAG-6a-Ataxin3Q80). Lentiviral particles were produced using VectorBuilder’s virus packaging service. WT MEFs or N2a cells were plated in 96-well plates at 50% confluency and transduced the following day with virus-containing complete medium supplemented with 10 μg/ml polybrene. After 24 h, the medium was replaced with fresh complete medium, and cells were selected with 5 μg/ml Blasticidin S Hydrochloride (FUJIFILM-Wako, Cat#026-18711) to establish stable cell lines. cDNA sequencing of the stable cell lines indicated that the expressed ATXN3 contains Q79 (79 glutamine residues).

### Immunoblotting

MEFs or N2a cells were solubilized in 1% Triton lysis buffer, centrifuged (15 000 × *g*, 10 min at 4°C), and the supernatant was used as cell lysate (Triton X-100 soluble fraction). To prepare the 1% Triton X-100 insoluble fraction, the pellet was washed and solubilized using 8 M urea in 3× sample buffer and sonicated. Cell lysates were mixed with 3× sample buffer. The samples were separated using SDS–polyacrylamide gel electrophoresis (SDS–PAGE) and transferred onto polyvinylidene difluoride (PVDF) membranes (Bio-Rad, Cat#1620177), which were then blocked with 5% skim milk (FUJIFILM-Wako Cat#190-12865) prepared in phosphate-buffered saline (PBS) containing 0.1% Tween 20 for 30 min at 25°C. This was followed by overnight incubation with primary antibodies prepared in 3% bovine serum albumin (BSA) in PBS at 4°C. After washing with 0.1% Tween 20 in PBS, the membranes were probed with horseradish peroxidase-conjugated secondary antibodies (Invitrogen, Cat#31430 or Cat#31460; DAKO, Cat#P0449). Signals were visualized using ImmunoStar Zeta (FUJIFILM-Wako, Cat#295-72404) or ImmunoStar LD (FUJIFILM-Wako, Cat#290-69904) and detected using the FluorChem Chemiluminescence Imaging System (Alpha Innotech) or FUSION chemiluminescence imaging system (Vilber-Lourmat). Antibodies used for immunoblotting were rabbit polyclonal anti-DHX8 (Bethyl Laboratories, Cat#A300-624A; 1:1000), rabbit polyclonal anti-eIF4A1 (Abcam, cat#ab31217; 1:1000), mouse monoclonal anti-β-actin (Sigma–Aldrich, Cat#A1978, Clone#AC-15; 1:10000), mouse monoclonal anti-GFP (Santa Cruz Biotechnology, Cat#sc-9996, Clone#B-2, 1:1000), rabbit monoclonal anti-LIMPII antibody (Abcam, Cat#ab176317, Clone#EPR12080, 1:1000), rabbit polyclonal anti-RAB5A (Santa Cruz Biotechnology, Cat#sc-309, 1:1000), mouse monoclonal anti-KDEL (Enzo Life Science, Cat#SPA-827, Clone#10C3, 1:1000), mouse monoclonal anti-COX4I1 (Elabscience, Cat#E-AB-22002, Clone#2E2, 1:1000), mouse monoclonal anti-GM130 (BD Transduction Laboratories, Cat#G65120, 1:1000), mouse monoclonal anti-Lamin A/C (Cell Signaling, Cat#4777, Clone#4C11, 1:1000) and goat polyclonal anti-GAPDH (Santa Cruz Biotechnology, Cat#sc-20357, 1:1000), mouse monoclonal anti-polyQ (Merck, Cat#MABN2427, Clone#MW1; 1:2000), and mouse monoclonal anti-DYKDDDDK (FLAG) tag (FUJIFILM-Wako, Cat#012-22384, Clone#1E6; 1:3000). A rabbit polyclonal antibody specific for the C-terminal region of SIDT2 (DLDTVQRDKIYVF) [10] was used at a dilution of 1:1000.

### RNA interference

MEFs were transfected with 10–20 nM small interfering RNAs (siRNAs). *Dhx8*, *Eif4a1*, or control siRNAs using Lipofectamine RNAiMAX (Invitrogen, Cat#13778150) for 48–72 h, according to the manufacturer’s instructions. The target sequences are 5′-GAA GAU GAG GAU CUU GAA A-3′ (*Dhx8* #1), 5′-GCA GAA AGU GUA CAA UUC U-3′ (*Dhx8* #2), 5′-CGA GAU UGU GGA UAG CUU U-3′ (*Eif4a1* #1), 5′-CAA ACA CAA AAU UCU GAA U-3′ (*Eif4a1* #2), 5′-GCC ACA ACG UCU AUA UCA U-3′ (*EGFP*; control), and 5′-UUC UCC GAA CGU GUC ACG U-3′ (universal negative control). The siRNA targeting the universal negative control was used as a non-targeting control when the EGFP-tagged plasmids were co-transfected.

### Endogenous RNA degradation assay (pulse-chase assay)

Endogenous total RNA degradation was measured as previously described [8–10]. For the knockdown experiments, MEFs were seeded at 5 × 10^4^ cells/well in 24-well plates (FALCON, Cat#353047) and transfected with siRNAs for 48 h. For overexpression experiments, N2a cells were seeded at 1.4 × 10^5^ cells/well in 12-well plates (FALCON, Cat#353043) and grown for 24 h, then co-transfected with pCI-neo-mSIDT2 (0.02 μg) and/or pCI-neo-DHX8 (0.02 μg) together with carrier DNA (pCI-neo empty vector) up to 0.2 μg DNA/well, using 1 mg/ml polyethylenimine solution (1 mg/ml polyethylenimine, 25 mM HEPES, pH 7, and 150 mM NaCl) (0.8 μl/well). At the appropriate post-transfection time (48 h for siRNA transfection or 4 h for plasmid transfection), 0.3 μCi/ml [^3^H]-uridine (PerkinElmer, Cat#NET367001MC) was added for RNA labeling. After 24 h of labeling, the cells were washed and cultured in growth medium containing 5 mM unlabeled uridine for 0 or 24 h (chase). The cells were then trypsinized and acid-insoluble radioactivity was measured using a liquid scintillation counter. In some experiments, chloroquine (50 μM) was added during the chase period. Radioactivity was calculated as the percentage of the acid-insoluble radioactivity at 0 h, and RNA degradation rate was calculated by subtracting the relative radioactivity at 24 h from 100%.

### Total RNA extraction

Total RNA was isolated from cells or mouse brains using TRI Reagent (Molecular Research Center, Cat#TR118) following the manufacturer's protocol, and the RNA concentration was determined with a NanoPhotometer® (Implen).

### RNA uptake assay by isolated lysosomes

Lysosome isolation and RNA uptake assays were performed as previously described [8, 10]. Lysosomes were isolated from 1.5 × 10^8^ MEFs transfected with control or *Dhx8* #1 siRNA (collected 72 h post-transfection) by density gradient centrifugation using a Lysosome Enrichment Kit for Tissues and Cultured Cells (Thermo Fisher Scientific, Cat#89 839), rinsed, and resuspended in 100 μl of 0.3 M sucrose. For each assay, 10 μl of the isolated lysosomes were incubated with 5 μg total RNA at 37°C for 3 min in 30 μl buffer (pH 7) containing 10 mM 3-(N-morpholino)propanesulfonic acid (MOPS), 0.3 M sucrose, and 10 mM ATP. After incubation, the reaction was stopped by incubation at 4°C, and lysosomes were removed by centrifugation. The remaining RNA in the supernatant was extracted using TRI Reagent. The extracted RNA was examined using agarose gel electrophoresis with ethidium bromide staining, followed by UV illumination and signal intensity quantification using a FUSION chemiluminescence imaging system (Vilber-Lourmat).

### Analysis of interaction between DHX8 and RNA within cells using UV crosslinking

pCI-neo-DHX8-HB or pCI-neo-DHX8-R620A-HB transfected N2a cells were crosslinked on ice with 254 nm UVC light at 300 mJ/cm^2^ for 5 min using a FUNA UV Crosslinker (FUNAKOSHI, Cat#FS-1500). Cells were solubilized in 1% iCLIP lysis buffer (50 mM Tris–HCl, pH 7.4, 100 mM NaCl, 1% Igepal CA-630, 0.1% SDS, and 0.5% sodium deoxycholate) [19]. Lysates were sonicated for 20 set (1 s ON and 1 s OFF) at 10% amplitude using a Handy Sonic (TOMY Cat#UR-21P) and analyzed using immunoblotting.

### Immunofluorescent staining

MEFs were seeded on coverslips on six-well plates (2.6 × 10^5^ cells/well), and after 24 h incubation, cells were transfected with 2.5 μg of pEGFP-N1-mSIDT2 or pEGFP-N1-LAMP1 using Lipofectamine LTX Reagent with PLUS Reagent (Invitrogen, Cat#15338100), according to the manufacturer’s instructions. N2a cells were seeded on coverslips on six-well plates (5 × 10^5^ cells/well), and after 24 h incubation, cells were transfected with 0.5 μg of pEGF-N1-mSIDT2 using 1 mg/ml polyethylenimine solution (2 μl/well). To observe the cytoplasmic RNA foci formed by HTTex1-eCAG repeats, MEFs were seeded on a six-well plate (1 × 10^5^ cells/well), and grown for 24 h, then cells were co-transfected with 1 μg pHR-tdMCP-YFP, 1 μg pHR-Tre3G-47 × CAG-12 × MS2, 1 μg pTet-Off Advanced Vector, and 1 μg pTagBFP-N-LAMP1 using Lipofectamine LTX Reagent with PLUS Reagent (Invitrogen) according to the manufacturer’s instructions. At 24 h post-transfection, cells were seeded onto coverslips in six-well plates (1.75 × 10^5^ cells/well) and transfected with siRNA against *Dhx8* (#1) or universal negative control siRNA using Lipofectamine RNAiMAX (Invitrogen) for 48 h, following the manufacturer’s protocol. In some experiments, 24 h post-transfection, cells were incubated at 37°C for an additional 24 h in culture medium supplemented with 20 nM bafilomycin A1 (BioViotica, Cat#BVT-0252-C100), a vacuolar ATPase inhibitor, to inhibit degradation via lysosomes. After 24 h post-transfection or immediately after bafilomycin A1-treatment, the cells were fixed for 20 min with 4% paraformaldehyde (FUJIFILM-Wako Cat#163-20145) at 25°C, and then permeabilized for 5 min with 0.1% Triton X-100 in PBS. After blocking for 30 min with 0.5% BSA in PBS at 25°C, the cells were incubated with the anti-DHX8 antibody (Bethyl Laboratories, Cat#A300-624A; 1:100) in 0.5% BSA in PBS for 1 h at 25°C, and then with Cy3 AffiniPure Donkey Anti-Rabbit IgG (H + L) (Jackson Immuno Research, Cat#711–165-152; 1:500) for 1 h at 25°C. The cells were rinsed with PBS before each step. Coverslips were mounted on slides with ProLong Gold antifade reagent with DAPI (Invitrogen, Cat#P36931), incubated for 18 h at 25°C, and image acquisition was performed using a confocal laser microscope (FV1000DIX81, Olympus) or SpinSR10 spinning disk confocal super-resolution microscope (Olympus). Line scan analysis was performed using ImageJ software (version 2.14.0/1.54f). Colocalization analysis of DHX8 and eCAGr RNA foci was performed manually using ImageJ by calculating the percentage of the number of eCAGr RNA foci that colocalized with DHX8 among the total number of observed eCAGr RNA foci (27 foci) in five randomly selected cells.

### Analysis of protein–protein interaction using pull-down assay

To examine the interaction between SIDT2 and DHX8 or its deletion mutants, transfected N2a cells were solubilized in 1% Triton lysis buffer, and the cell lysates were incubated with Streptavidin Sepharose High Performance beads (GE Healthcare, Cat#17-5113-01) for 2 h at 4°C. After washing four times with 1% Triton lysis buffer, the proteins were eluted in urea elution buffer (8 M urea, 2% SDS, 3 mM biotin) at 30°C for 30 min, and analyzed using immunoblotting.

### Quantitative PCR

After total RNA extraction, cDNA was synthesized using a PrimeScript RT Reagent Kit with gDNA Eraser (Perfect Real Time) (Takara, Cat#RR047A) according to the manufacturer’s protocol. *HTTex1*-*EGFP*, *ATXN3* (Q79), and *Gapdh* mRNA levels were quantified by quantitative PCR (qPCR) using a CFX96^™^ Real-Time System (Bio-Rad) with Luna Universal qPCR Master Mix (New England Biolabs, Cat#M3003E). The following primers were used: *EGFP* forward primer, 5′-GTAAACGGCCACAAGTTCAGCGTG-3′; *EGFP* reverse primer, 5′-AAGTCGTGCTGCTTCATGTGGTCG-3′; *ATXN3* (Q79) forward primer, 5′-CAAAAAAGCAGGCTGCCACCATG-3′; *ATXN3* (Q79) reverse primer, 5′-ATCCATATTTCCAGAAGGCTGCTG-3′; mouse *Gapdh* forward primer (Set 1), 5′-TGTGTCCGTCGTGGATCTGA-3′; mouse *Gapdh* reverse primer (Set 1), 5′-TTGCTGTTGAAGTCGCAGGAG-3′; mouse *Gapdh* forward primer (Set 2), 5′-TGTCAAGCTCTTTCCTGGTATG-3′; mouse *Gapdh* reverse primer (Set 2), 5′-TTATGGGGGTCTGGGATGGA-3′. *HTTex1*-*EGFP* levels were normalized to *Gapdh* using Set 1, and *ATXN3* levels were normalized using Set 2.

### HTT mRNA degradation assay

MEFs were seeded at 6 × 10^5^ cells in 6 cm dish (FALCON, Cat#353002), grown for 24 h, then co-transfected with 2 μg pTet-Off, 2 μg pTRE-Tight-*HTTex1-*CAG*-*22 or 145-*EGFP* together with carrier DNA [pCI-neo empty vector up to 6 μg DNA/well using Lipofectamine LTX Reagent with PLUS Reagent (Invitrogen)], according to the manufacturer’s instructions. At 24 h post-transfection, MEFs were seeded into 24-well plates and transfected with *Dhx8* #1 siRNA or a universal negative control siRNA using Lipofectamine RNAiMAX (Invitrogen) for 48 h. Then, 1 μg/ml doxycycline (Clontech, Cat#631311) was added to suppress the transcription of *HTTex1*-*EGFP* gene and after 6 h cells were harvested, and total RNA was extracted using TRI Reagent. The expression levels of *HTTex1*-*EGFP* mRNA were quantified by qPCR. Results are shown as percentage of RNA degradation, which was calculated by subtracting the relative mRNA levels at 6 h (percentage at 0 h) from 100%.

### Live-cell imaging

MEFs were seeded at 1 × 10^5^ cells/well on six-well plate, and grown for 24 h, then cells were co-transfected with 1.5 μg pHR-tdMCP-YFP, 1.5 μg pHR-Tre3G-47 × CAG-12 × MS2, and 1 μg pTet-Off Advanced Vector using Lipofectamine LTX Reagent with PLUS Reagent (Invitrogen) as instructed. At 24 h post-transfection, MEFs were split to μ-Dishes 35-mm (Ibidi, Cat#81156), and were transfected with *Dhx8* #1 siRNA or universal negative control siRNA using Lipofectamine RNAiMAX (Invitrogen). After 48 h post-transfection, lysosomes were stained with LysoTracker Red DND-99 (Invitrogen, Cat#L7528), according to the manufacturer’s instructions. The cells were examined using a Spinsr10 spinning disk confocal super-resolution microscope (Olympus).

### Statistical analyses

Statistical analyses were performed using data from biological replicates, with the exception of Fig. 6D. The number of biological replicates (*n*) is indicated in the figure legends or figure panels. In Fig. 6D, *n* indicates technical replicates (multiple cells analyzed from two independently cultured dishes). Statistical analyses were conducted using the Student’s *t*-test for comparisons between two groups. Tukey’s or Dunnett’s multiple comparison tests were used for comparisons involving more than two groups.

## Results

### Comprehensive analysis of proteins binding to consecutive sequences of guanine using a pull-down assay

**Figure 1.**
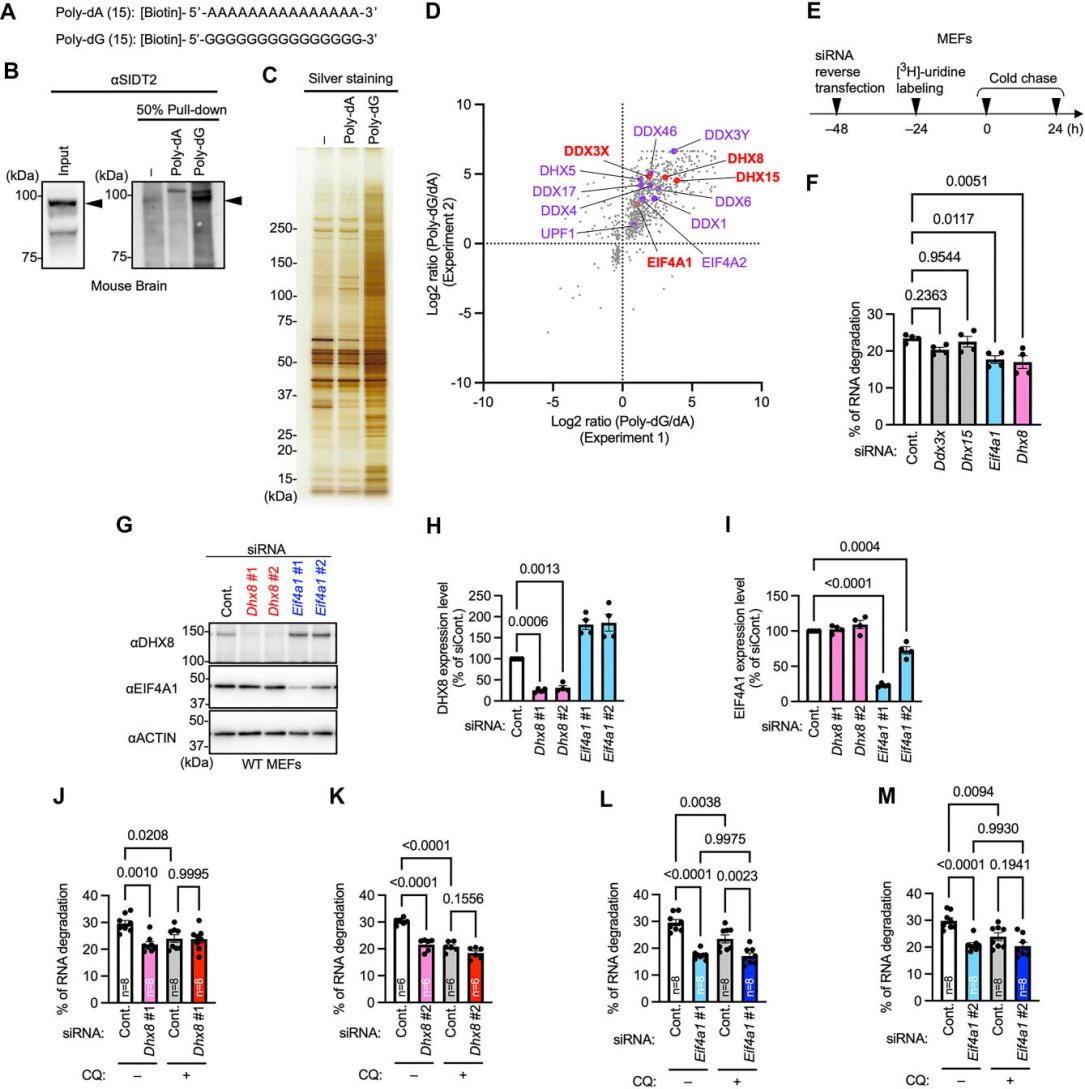
Screening of RNA helicases that are involved in RNA degradation via lysosome. (**A**) Oligonucleotide DNA sequences used for pull-down assays. (**B**and**C**) Interaction of the endogenous proteins with poly-dA (15-mer) or poly-dG (15-mer) in mouse brain lysates. Pull-down assays were performed using oligo DNA (1 nmol). Interacting SIDT2 was detected by immunoblotting (B). Silver stain analysis of the interacting proteins (C). (**D**) Interaction proteins were identified using LC/MS-MS analysis. Scatterplot of the proteins for which there was a significant difference in binding to poly-dG and poly-dA in both of two independent experiments. Data represent the log2 of the abundance ratio of proteins interacting with poly-dG to that with poly-dA. RNA helicases which were reported to be present on lysosomes or not were indicated red or purple, respectively. (**E**) Schematic of the experimental method for measuring intracellular degradation of endogenous RNA in MEFs transfected with siRNAs. Cells were labeled with [^3^H] uridine, grown in medium with unlabeled uridine, and chased for 24 h. Radioactivity was expressed as a percentage of degraded RNA. (**F**) RNA degradation in MEFs transfected with indicated siRNAs. Data are mean ± SEM (*n* = 4). (**G–I**) Levels of DHX8 and EIF4A1 proteins in MEFs transfected with indicated siRNA were analyzed using immunoblotting. Data are mean ± SEM (*n* = 4). (**J–M**) RNA degradation in MEFs transfected with indicated siRNAs and treated with or without CQ (50 μM). Data are mean ± SEM. Numbers within the bars indicate *n*. *P*-values are from Dunnett’s multiple comparisons test (F, H, and I) or Tukey’s multiple comparisons test (J–M); CQ, chloroquine.

We aimed to identify helicases that specifically bind to poly-G/dG by conducting a comprehensive analysis of the proteins that interact with biotinylated poly-dG (Fig. 1A). We used 15-mer poly-dG, the length of which is sufficient to be selectively incorporated into lysosomes via RNautophagy and to bind to the cytoplasmic sequences of LAMP2C and SIDT2 *in vitro* [7, 9] (discussed later). Poly-dA (15-mer) was used as a negative control (Fig. 1A) because LAMP2C and SIDT2 do not bind to poly-A/dA (15-mers) [7, 9]. Pull-down assays were performed using mouse brain lysates and these oligonucleotides. The results showed that endogenous SIDT2 interacts with poly-dG (Fig. 1B and Supplementary Fig. S1A). Silver staining revealed that poly-dG bound more proteins than poly-dA (Fig. 1C). LC-MS/MS analyses of these proteins revealed 917 proteins which significantly interacted with poly-dG compared to poly-dA, and vice versa, in two independent experiments (Fig. 1D, and Supplementary Tables S1 and S2). The identified proteins included 13 RNA helicases (Fig. 1D). Among these proteins, we focused on DDX3X, DHX15, EIF4A1, and DHX8, which are reported to be present in lysosomes [20].

### *Dhx8* or *Eif4a1* knockdown impairs cellular RNA degradation

We investigated the effects of *Ddx3x*, *Dhx15*, *Eif4a1*, and *Dhx8* on RNA degradation in MEF, using a pulse-chase assay (Fig. 1E and Supplementary Fig. S1B). Our results showed that RNA degradation was significantly impaired in cells knocked down with *Eif4a1* or *Dhx8* compared to that in control cells but not in cells knocked down with *Ddx3x* or *Dhx15* (Fig. 1F–I and Supplementary Fig. S2A). Similar results were obtained with other siRNAs against *Eif4a1* or *Dhx8* (Fig. 1G–M and Supplementary Fig. S2B–E). To assess whether the effect of *Eif4a1* or *Dhx8* knockdown on RNA degradation was due to an impaired lysosomal degradation pathway, we conducted pulse-chase experiments using chloroquine (CQ), which inhibits lysosomal hydrolases. Treatment of control siRNA-transfected cells with CQ inhibited RNA degradation (Fig. 1J–M). Comparison between the CQ-treated control and *Dhx8* siRNA-transfected cells showed no significant differences (Fig. 1J and K). These results suggest that the knockdown of *Dhx8* impairs lysosomal RNA degradation. In contrast, CQ-treated *Eif4a1-*knockdown cells exhibited further inhibition of RNA degradation compared to CQ-treated control siRNA-transfected cells (Fig. 1L). RNA degradation in CQ-treated *Eif4a1* knockdown cells was not significantly different from that in CQ-untreated *Eif4a1-*knockdown cells (Fig. 1L and M). These results suggest that EIF4A1 is involved in both lysosomal and cytoplasmic RNA degradation pathways.

### Pathway involved in DHX8-mediated RNA degradation is SIDT2-dependent pathway not macroautophagy

**Figure 2.**
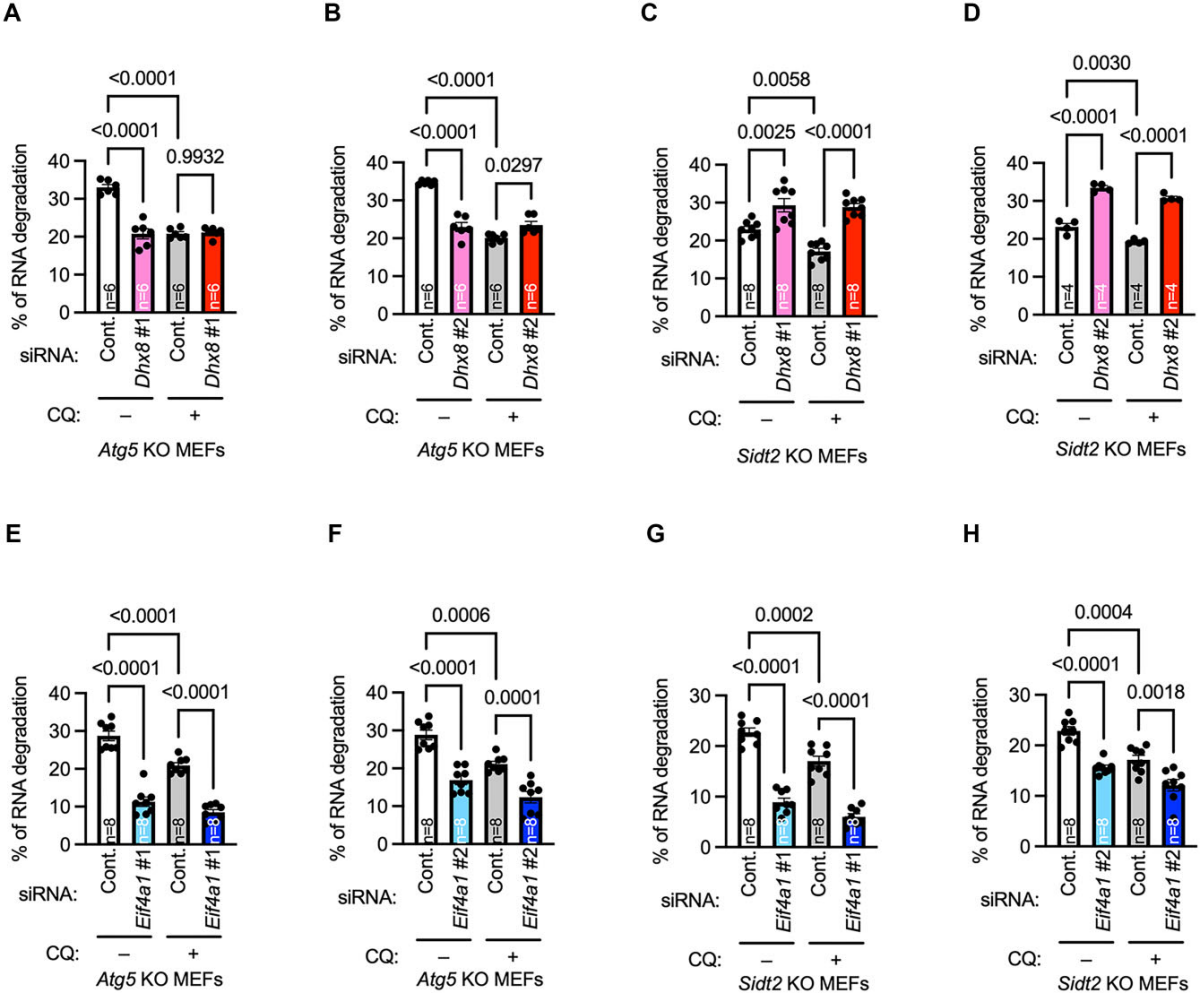
Pathway involved in DHX8-mediated RNA degradation is SIDT2-dependent pathway, not macroautophagy. (**A–H**) RNA degradation in *Atg5* KO (A, B, E, and F) or *Sidt2* KO (C, D, G, and H) MEFs transfected with indicated siRNAs and treated with or without CQ (50 μM). Data are mean ± SEM. Numbers within the bars indicate *n*. *P*-values are from Tukey’s multiple comparisons test (A–H).

To investigate the involvement of macroautophagy, we performed pulse-chase experiments using macroautophagy-deficient *Atg5* KO MEFs. Our results showed that RNA degradation was impaired in *Dhx8*-knockdown cells compared to control siRNA-transfected cells (Fig. 2A and B, and Supplementary Figs S2F, G, and S3A–C). Furthermore, the treatment of control siRNA-transfected cells with CQ inhibited RNA degradation (Fig. 2A and B), whereas *Dhx8* knockdown did not exhibit an additional inhibitory effect on CQ-treated cells (Fig. 2A and B). RNA degradation was also impaired by *Dhx8*-knockdown in *Atg13* KO MEFs (Supplementary Figs S2N and S3D). These results indicated that DHX8 is involved in lysosomal degradation pathways other than macroautophagy.

To investigate the involvement of RNautophagy, we performed pulsed-chase experiments using *Sidt2* KO MEFs. Notably, *Dhx8* knockdown did not inhibit (did enhance, discussed later) RNA degradation in CQ-untreated or -treated cells (Fig. 2C and D, and Supplementary Figs S2H, I, and S3E–G). These results suggest that DHX8 is involved in RNA degradation via SIDT2-dependent RNautophagy.

*Eif4a1* knockdown inhibited RNA degradation in both *Atg5 or Sidt2* KO MEFs (Fig. 2E–H and Supplementary Fig. S2J–M), suggesting that EIF4A1 is involved in an RNA degradation pathway that is independent of macroautophagy and RNautophagy. Therefore, we focused on the specific role of DHX8 in lysosomal RNA degradation.

### Knockdown of Dhx8 impairs RNA uptake in isolated lysosomes

**Figure 3.**
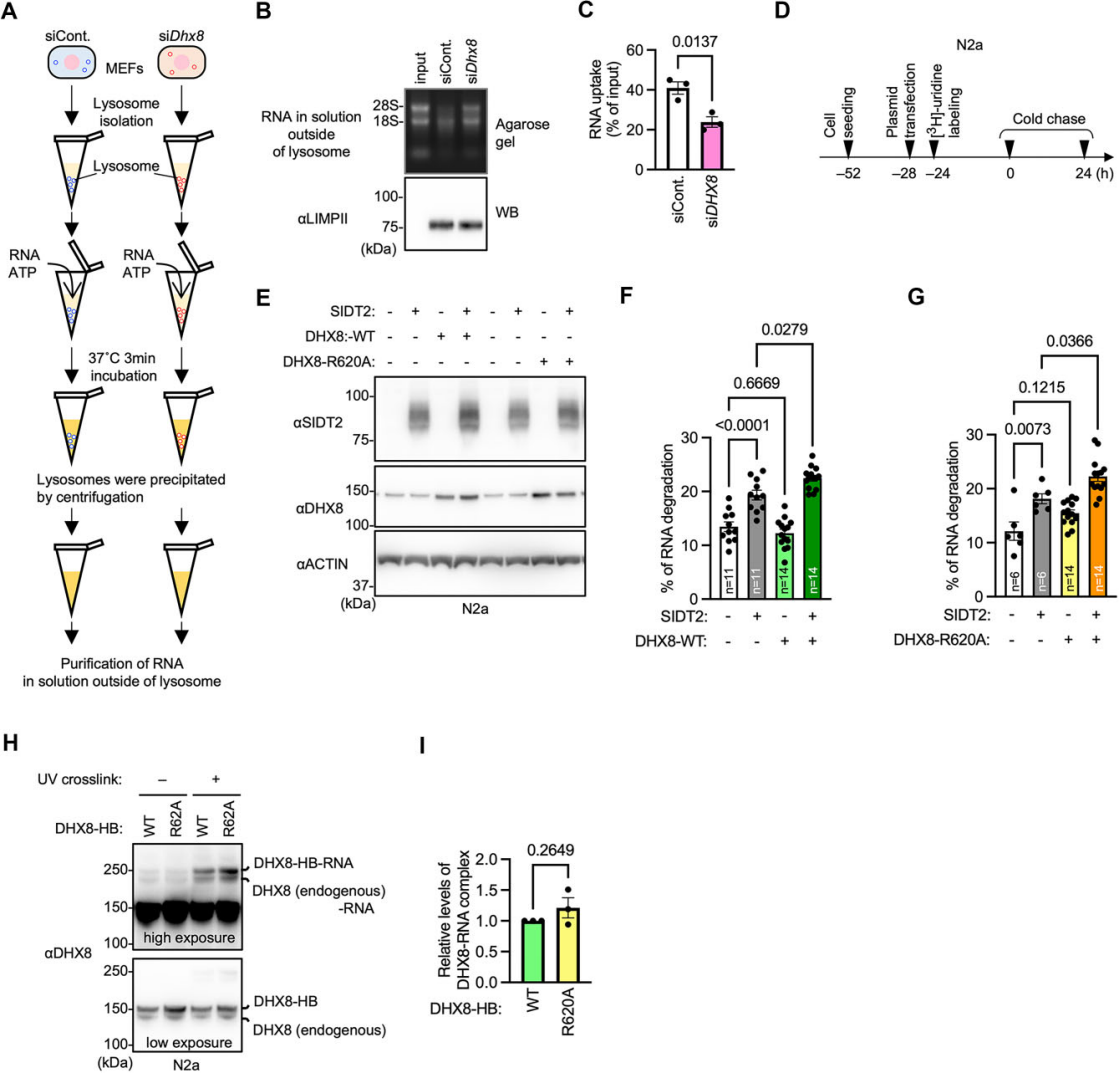
DHX8 mediates RNautophagy and cooperates with SIDT2 independent of enzymatic activity of DHX8. (**A–C**) Schematic of *in vitro* RNA uptake assay using isolated lysosomes (A). MEFs were transfected with control or *Dhx8* siRNA. After 72 h, lysosomes were isolated by density gradient ultracentrifugation. Lysosomes were incubated with 5 μg total RNA (purified from mouse brains) for 3 min at 37°C in the presence of ATP, and then lysosomes were precipitated by centrifugation, and RNA levels in the solution outside the lysosomes were measured (B). RNA uptake levels were calculated by subtracting the RNA levels in the solution outside the lysosomes from the RNA input levels (C). Data are mean ± SEM (*n* = 3). (**D**) Schematic of the experimental method for measuring intracellular degradation of endogenous RNA in N2a cells overexpressing SIDT2 and/or DHX8. (**E**) Overexpression of SIDT2 and/or DHX8 (WT or R620A) in N2a cells was confirmed using immunoblotting. (**F** and **G**) RNA degradation in N2a cells overexpressing indicated proteins. Data are mean ± SEM. Numbers within the bars indicate *n*. (**H**and **I**) N2a cells were overexpressed with DHX8–HB or DHX8–R620A–HB and were UV crosslinked. The DHX8 and covalently crosslinked DHX8–RNA complex were separated by SDS–PAGE and detected using immunoblotting (H). The DHX8–RNA complex levels were normalized by the DHX8–HB levels (I). Data are mean ± SEM (*n* = 3). *P*-values are from unpaired *t* test (C and I) or Tukey’s multiple comparisons test (F and G).

To further examine the effects of *Dhx8* knockdown on RNautophagy activity, we transfected WT MEFs with siRNAs targeting *Dhx8*. We then isolated the lysosomes (Supplementary Fig. S4) and performed RNA uptake assays (Fig. 3A and Supplementary Fig. S4). Lysosomes from *Dhx8*-knockdown cells showed significantly reduced RNA uptake compared to those from control siRNA-transfected cells (Fig. 3B and C). Collectively, these results indicated that the pathway involved in DHX8-mediated RNA degradation is RNautophagy.

### DHX8 cooperates with SIDT2 in RNautophagy independent of enzymatic activity of DHX8

An *in vitro* study showed that the substitution of arginine at position 620 with alanine (R620A) in DHX8 reduced its ATP- and RNA-binding affinity, attenuated RNA-stimulated ATPase activity, and compromised nucleic acid duplex unwinding activity [21]. To elucidate whether the helicase activity of DHX8 is implicated in RNautophagy, we overexpressed SIDT2 along with either the WT DHX8 (DHX8–WT) or the DHX8–R620A mutant in Neuro2a (N2a) cells and performed a pulse-chase assay (Fig. 3D and E). We used N2a cells because we were able to detect increased RNA degradation by overexpressing SIDT2 [10]. RNA degradation was significantly increased in cells overexpressing SIDT2 compared to that in control cells (Fig. 3F and G, Supplementary Fig. S2O and P). In contrast, the overexpression of DHX8–WT or DHX8–R620A did not increase RNA degradation (Fig. 3F and G). Notably, the co-expression of SIDT2 with either DHX8–WT or DHX8–R620A significantly promoted RNA degradation compared to the expression of SIDT2 alone (Fig. 3F and G), suggesting that DHX8 functions as an adapter for RNA during RNautophagy. Therefore, we compared the RNA-binding activities of DHX8–WT and –R620A in cells. The results showed that RNA-binding activity of DHX8–R620A was comparable to that of DHX8–WT (Fig. 3H and I). These results indicate that DHX8 functions cooperatively with SIDT2 during RNautophagy and suggest that the function is dependent on the RNA-binding activity of DHX8, but independent of enzymatic activity.

### DHX8 interacts with SIDT2 and partly localizes to the cytoplasmic side of the lysosomal membrane

**Figure 4.**
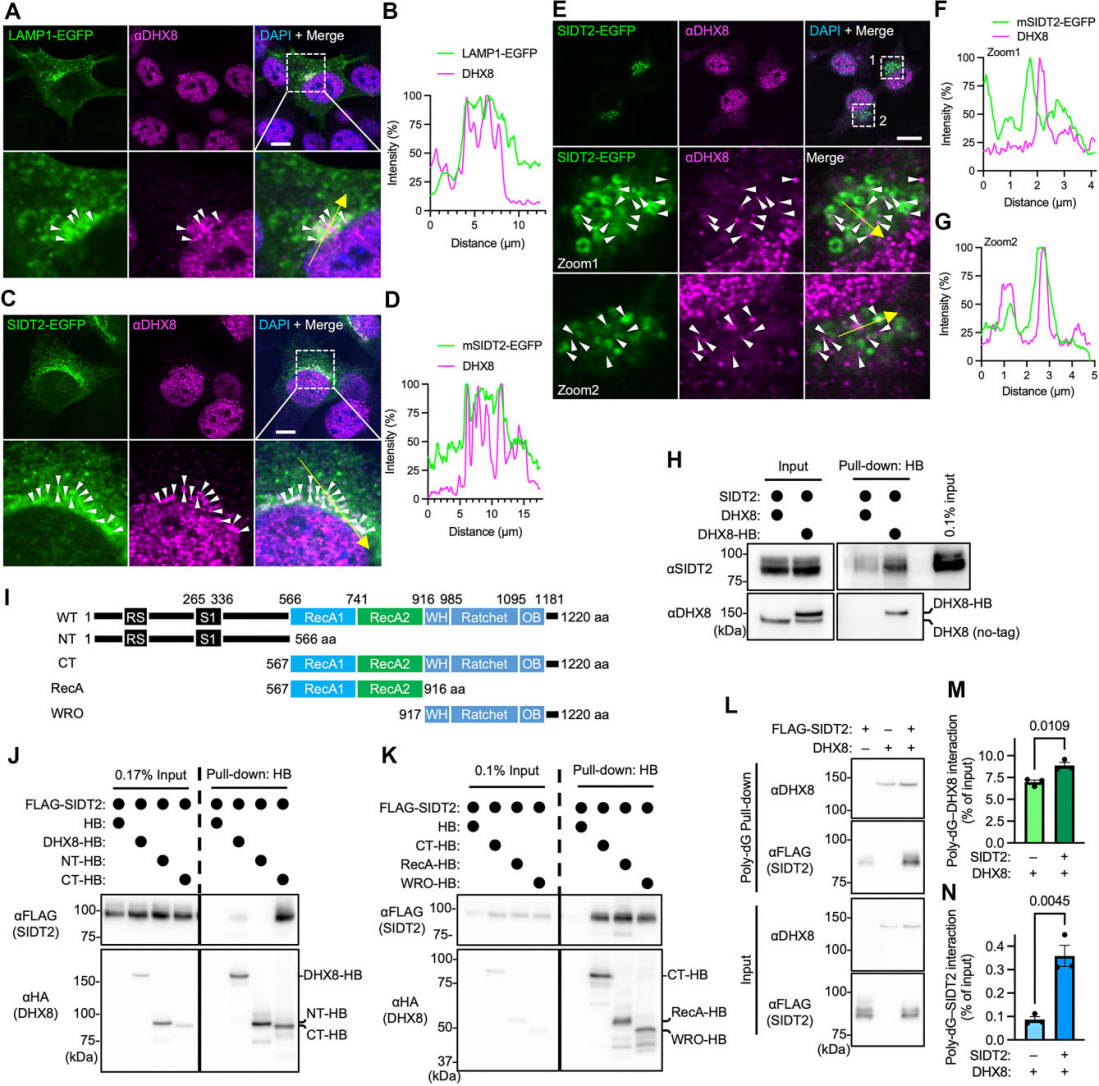
DHX8 interacts with SIDT2 and partly localizes to the cytoplasmic side of the lysosomal membrane. (**A**and **B**) Cellular localization of endogenous DHX8 and LAMP1–EGFP in MEFs (A). Boxed regions are enlarged (A, lower panels); scale bar: 10 μm. Line scans show profiles of fluorescence intensity against line distance (B). (**C** and **D**) Cellular localization of endogenous DHX8 and SIDT2–EGFP in MEFs (C). Boxed regions are enlarged (A, C, and E, lower panels); scale bar: 10 μm. Line scans (D). (**E–G**) Cellular localization of endogenous DHX8 and SIDT2–EGFP in N2a cells (E). Boxed regions are enlarged (E, lower panels); scale bar: 10 μm. Line scans (F and G). (**H**) N2a cells overexpressing SIDT2 and DHX8–HB or untagged DHX8 were subjected to streptavidin (SA) pull-down assays. Interacting proteins were detected using immunoblotting. (**I**) Schematic representations of the full-length (WT) and truncated DHX8 constructs are shown with their respective domain compositions: NT (N-terminal fragment containing RS and S1 domains), CT (C-terminal fragment containing RecA1, RecA2, WH, Ratchet, and OB domains), RecA (fragment containing only RecA1 and RecA2 domains), and WRO (fragment containing WH, Ratchet, and OB domains). RS, arginine/serine-rich domain; S1, S1 domain; RecA1, RecA-like domain 1; RecA2, RecA-like domain 2; WH, winged helix domain; Ratchet, ratchet domain; OB, oligonucleotide/oligosaccharide-binding fold. Numbers indicate amino acid positions. (**J**and **K**) N2a cells overexpressing SIDT2–FLAG and indicated DHX8–HB variants were subjected to streptavidin (SA) pull-down assays. Interacting proteins were detected using immunoblotting. (**L–** **N**) Simultaneous interaction of the overexpressed SIDT2–FLAG and DHX8 with oligonucleotides in N2a lysates. Pull-down assays were performed using 1 nmol of poly-dG (15-mer). Interacting proteins were detected by immunoblotting. Data are mean ± SEM (*n* = 3). *P*-values are from unpaired *t* test (**M** and **N**).

To further investigate the relationship between DHX8 and SIDT2, we examined their localization in MEFs and N2a cells. SIDT2 localizes mainly to the lysosomes [8, 20]. Most of the immunofluorescent DHX8 signal was co-localized with DAPI-stained nuclei (Fig. 4A). Analysis of the lysosomal organelle marker LAMP1–EGFP revealed that DHX8 was partially co-localized with LAMP1 in MEFs (Fig. 4A and B). DHX8 was also partially co-localized with SIDT2–EGFP in MEFs (Fig. 4C and D). These results indicated that DHX8 partly co-localizes with SIDT2 in lysosomes. Analysis of N2a cells demonstrated that DHX8 was localized in proximity to SIDT2–EGFP (Fig. 4E and F) or co-localized with SIDT2–EGFP (Fig. 4E and G). Next, we examined the interaction between DHX8 and SIDT2 using a pull-down assay, which revealed the interaction (Fig. 4H). To identify the region of DHX8 that interacts with SIDT2, we performed pull-down assays using HB-tagged truncation mutants (Fig. 4I). As shown in Fig. 4J, the N-terminal fragment (residues 1–566) did not bind to SIDT2, whereas the C-terminal fragment (residues 567–1220) did. We then subdivided the C-terminal region into two fragments: residues 567–916 and 917–1220. Both fragments interacted with SIDT2 (Fig. 4K), suggesting that SIDT2 binds to the C-terminal portion of DHX8. To further investigate how SIDT2 and DHX8 interact with nucleic acids, we performed pull-down assays using poly-dG (15-mer) as a model substrate. When expressed individually, both SIDT2 and DHX8 bound to poly-dG (Fig. 4L). Notably, co-expression of both proteins enhanced poly-dG binding for each other (Fig. 4L–N), suggesting a cooperative effect rather than mutual competition. These results suggest that SIDT2, DHX8, and nucleic acids may form a ternary complex. Thus, DHX8 on the lysosomal membrane may enhance SIDT2-dependent RNautophagy, possibly via cooperative binding to nucleic acid substrates.

### DHX8 mediates degradation of CAG repeat RNA

**Figure 5.**
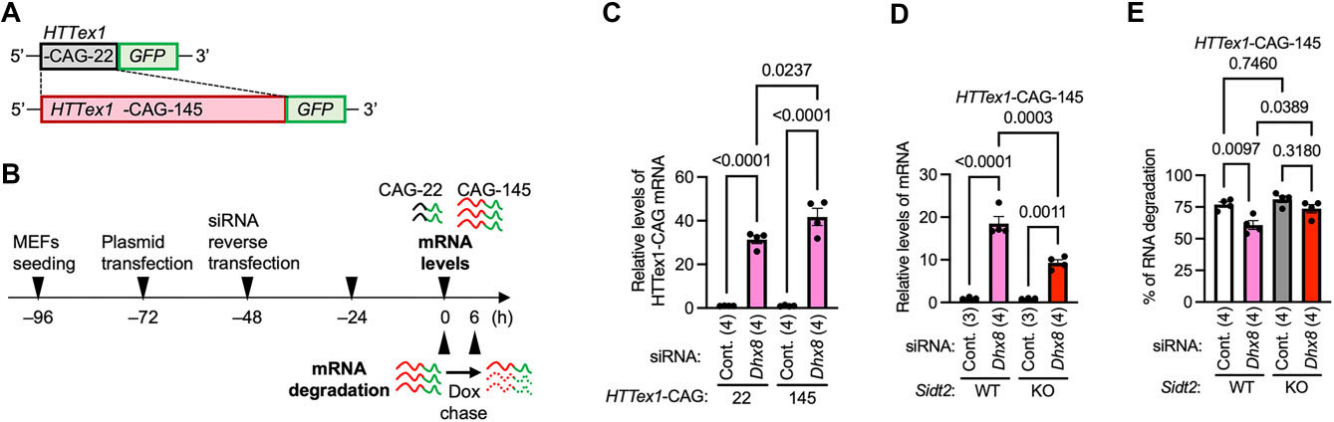
DHX8 mediates degradation of CAG repeat RNA. (**A**) Schematic representation of the constructs used in the experiment: *HTTex1* with CAG-22 repeats (WT) tagged with EGFP, *HTTex1* with CAG-145 repeats (mutant associated with Huntington’s disease) tagged with EGFP. (**B**) Experimental scheme for measuring mRNA levels and mRNA degradation of *HTTex1* constructs using Tet-Off system. MEFs were seeded at –96 h time point, followed by plasmid transfection at the –72 h time point and siRNA transfection at the –48 h time point. mRNA levels of *HTTex1* constructs were assessed at 0 h time point using qPCR. For mRNA degradation measurements, transcription of the constructs was inhibited by adding Dox at 0 h, mRNA levels of *HTTex1* constructs were assessed at 0 and 6 h, and RNA degradation (%) was calculated as described in “Materials and methods” section. (**C**) MEFs were transfected with *HTTex1* constructs, and then *Dhx8* was knocked down using siRNA. The mRNA levels of *HTTex1*–CAG-22–*EGFP* or *HTTex1*–CAG-145–*EGFP* were measured. (**D**and **E**) WT or *Sidt2* KO MEFs were transfected with expression vectors for *HTTex1*–145–*EGFP*, and then *Dhx8* was knocked down using siRNA. The mRNA levels (D) and mRNA degradation (E) of *HTTex1*–145–*EGFP* were measured. Data are mean ± SEM. Numbers in parentheses indicate *n*. *P*-values are from Tukey’s multiple comparisons test.

We have previously reported that RNautophagy degrades CAG repeat RNA [9, 13]. To understand the potential pathophysiological implications of DHX8-mediated RNA degradation, we examined whether DHX8 regulates the levels and degradation of CAG repeat RNA. We focused on the huntingtin (*HTT*) gene in which an eCAGr in exon 1 causes Huntington’s disease, a progressive neurodegenerative disorder. It is believed that eCAGr lead to neurodegeneration mainly through the production of expanded polyglutamine (polyQ)-containing cytotoxic mutant proteins [22]. CAG repeat RNAs and their RNA foci have also been suggested to contribute to toxicity [13, 23, 24]. In this study, we used a model containing exon 1 of the HTT gene (HTTex1), which is commonly used in polyQ diseases research [25]. WT or mutant *HTTex1* constructs containing 22 (*HTTex1*-CAG-22) or 145 (*HTTex1*-CAG-145) CAG repeats (Fig. 5A) and *Dhx8* siRNA were cotransfected into MEFs, and the effects of *Dhx8* knockdown on *HTTex1* mRNA levels were examined (Fig. 5B). Both CAG-22 and CAG-145 mRNA levels markedly increased upon *Dhx8* knockdown (Fig. 5C), and the level of CAG-145 mRNA was higher than that of CAG-22 (Fig. 5C), suggesting that DHX8 regulates CAG repeat RNA levels in a repeat length-dependent manner.

Next, we investigated whether the regulation of *HTTex1*- repeat RNA levels by DHX8 was SIDT2-dependent. Although *Dhx8* knockdown increased *HTTex1*–145 mRNA levels in *Sidt2* KO MEFs, the level of *Dhx8* knockdown *Sidt2* KO MEFs was significantly lower than that in *Dhx8* knocked-down WT MEFs (Fig. 5D).

We investigated the effect of *Dhx8* knockdown on the degradation of *HTTex1*–145 mRNA by using the Tet-off system (Fig. 5B). *Dhx8* knockdown reduced the degradation of *HTTex1*–145 mRNA in WT MEFs (Fig. 5E). In contrast, in *Sidt2* KO MEFs, *Dhx8* knockdown did not decrease the degradation of *HTTex1*–CAG-145 mRNA (Fig. 5E). Thus, DHX8 mediates the degradation of CAG repeat RNA in a SIDT2-dependent manner.

### DHX8 and SIDT2 cooperate in the clearance of cytoplasmic RNA foci of *HTTex1*–eCAGr

**Figure 6.**
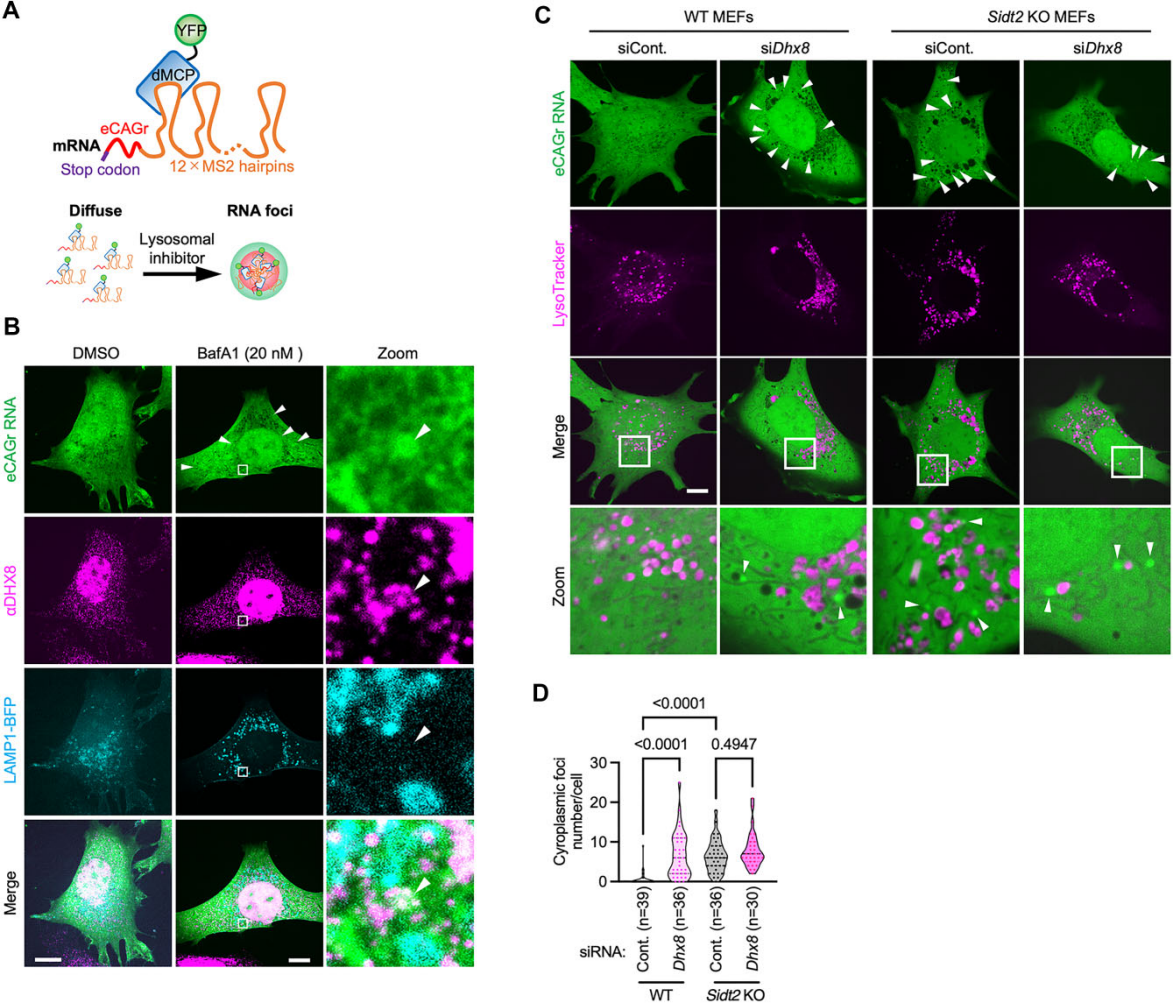
DHX8 and SIDT2 cooperate in the clearance of cytoplasmic RNA foci of HTTex1–eCAGr. (**A**) Schematic representation of the experimental design to visualize mRNA localization of CAG-47 repeat (eCAGr) construct. The construct contains an eCAGr region followed by 12 × MS2 hairpins. The YFP-dMCP binds to the MS2 hairpins, allowing visualization of mRNA, and therefore localization and formation of RNA foci can be assessed. (**B**) Cellular localization of eCAGr mRNA, endogenous DHX8, and LAMP1–BFP in MEFs treated with or without Bafilomycin A1 (20 nM). Arrowheads indicate eCAGr RNA foci. Boxed regions are enlarged (right panels). Representative confocal images are shown; scale bar: 10 μm. (**C** and **D**) Imaging of eCAGr mRNA in WT or *Sidt2* KO MEFs transfected with control or *Dhx8* siRNAs (C). Lysosomes were stained with LysoTracker Red. Arrowheads indicate eCAGr RNA foci (C). Boxed regions are enlarged (C, lower panels). Representative confocal images are shown; scale bar: 10 μm. The number of cytoplasmic foci per cell was counted and shown as truncated violin plots with median and quartiles (D). *P*-values are from Tukey’s multiple comparisons test (D).

Recently, eCAGr was reported to form RNA foci upon inhibition of degradation via lysosomes (bafilomycin A1, CQ, or NH_4_Cl) or knockdown of the RNautophagy receptor LAMP2C [13]. We examined the implications of DHX8 and SIDT2 in the formation of RNA foci of eCAGr mRNA using confocal laser microscopy. We confirmed that bafilomycin A1 treatment induced the formation of eCAGr RNA foci in MEFs (Fig. 6A and B, and Supplementary Fig. S5). Additionally, ∼92.6% of DHX8 co-localized with the RNA foci (Fig. 6B). We found that *Dhx8* knockdown induced the formation of RNA foci in WT MEFs (Fig. 6C, D, and Supplementary Fig. S6). RNA foci were also formed in *Sidt2* KO MEFs compared to WT MEFs (Fig. 6C, D, and Supplementary Fig. S6). Moreover, there was no difference in the number of RNA foci between WT MEFs transfected with *Dhx8* siRNA and *Sidt2* KO MEFs transfected with either control or *Dhx8* siRNA (Fig. 6C, D, and Supplementary Fig. S6). These findings suggest that DHX8 and SIDT2 cooperate to clear cytoplasmic RNA foci of eCAGr.

### DHX8 knockdown increases both soluble and insoluble polyQ protein levels in polyQ disease models

**Figure 7.**
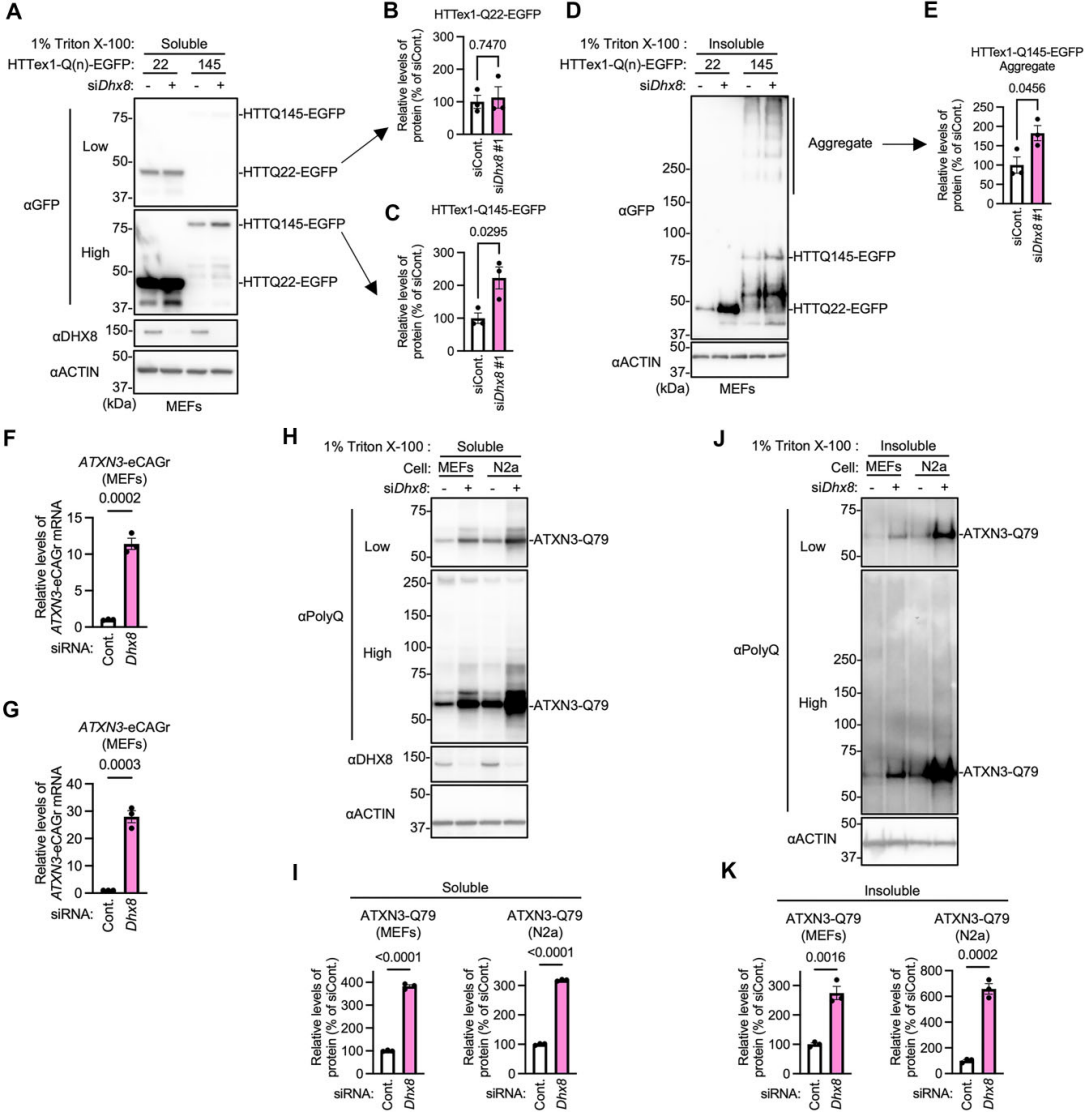
DHX8 knockdown increases both soluble and insoluble expanded polyQ proteins. (**A–E**) MEFs overexpressing HTTex1–Q22–EGFP or HTTex1–Q145–EGFP were transfected with control or *Dhx8* siRNAs. The cells were harvested and fractionated into 1% Triton X-100-soluble (A–C) and -insoluble (D and E) fractions, and the samples were analyzed using immunoblotting. Relative levels of soluble HTTex1-Q22 (B), 145-EGFP (C), and insoluble HTTex1-145-EGFP high molecular weight aggregates (E) were quantified. Data are mean ± SEM (*n* = 3). (**F**and **G**) MEFs (F) and N2a cells (G), both stably expressing ATXN3-Q79 were transfected with control or *Dhx8* siRNAs and incubated for 72 h. *ATXN3*-eCAGr mRNA levels were then measured. Data are mean ± SEM (*n* = 3). (**H–K**) MEFs and N2a cells stably expressing ATXN3-Q79 were transfected with control or *Dhx8* siRNA and incubated for 72 h. The cells were harvested and fractionated into 1% Triton X-100-soluble (H and I) and -insoluble (J and K) fractions, and the samples were analyzed using immunoblotting. Relative levels of soluble ATXN3–Q79 (I) and insoluble ATXN3–Q79 (K) were quantified. Data are mean ± SEM (*n* = 3). *P*-values are from unpaired *t* test.

We also investigated the effects of *Dhx8* knockdown on HTTex1 proteins containing a tract of 22 or 145 glutamines (HTTex1–Q22 or –Q145). It is widely accepted that mutant proteins with expanded polyQ, such as mutant HTT, exert cytotoxic effects through gain-of-function mechanisms [22]. In cells, mutant HTT forms insoluble aggregates, which are correlated with toxicity in cell models, as well as pathological changes in animal models of Huntington’s disease [26, 27]. Therefore, we analyzed the levels of both 1% Triton X-100-soluble proteins and the insoluble aggregates. We transfected MEFs with HTTex1–Q(*n*)–EGFP and either control or *Dhx8* siRNA, and analyzed the levels of soluble HTTex1–Q(*n*)–EGFP. We found that while *Dhx8* knockdown did not change the levels of soluble HTTex1–Q22–EGFP, it significantly increased the levels of soluble HTTex1–Q145–EGFP (Fig. 7A–C and Supplementary Fig. S7). Next, we analyzed the levels of insoluble HTTex1–Q145 high molecular weight aggregates and observed that *Dhx8* knockdown significantly increased the levels of these aggregates (Fig. 7D and E).

To further validate the generality of DHX8 function, we established and used MEF and N2a cell lines stably expressing full-length ATXN3–Q79, a polyQ disease-associated protein encoded by *ATXN3*, mutations of which cause spinocerebellar ataxia type 3. DHX8 knockdown significantly increased *ATXN3*–eCAGr mRNA levels in both cell types, as assessed by qPCR (Fig. 7F and G). Immunoblotting further revealed elevated levels of both soluble and insoluble ATXN3–Q79 proteins upon DHX8 knockdown (Fig. 7H–K).

## Discussion

In this study, we found that DHX8 regulates RNA degradation by the SIDT2-dependent RNautophagy pathway. DHX8 was partly localized to the cytoplasmic side of the lysosomal membrane and partly co-localized with SIDT2 (Fig. 4A–G). A pull-down assay showed that DHX8 interacted with SIDT2 in cells (Fig. 4H), although it was unclear whether this interaction was direct or indirect. The results of the experiments using the mutant DHX8–R620A suggested that the function of DHX8 is dependent on its RNA-binding activity (Fig. 3F–I). Taken together, these findings suggest that DHX8 functions as an adapter for RNA on the lysosomal membrane during RNautophagy. It is also possible that DHX8 recruits the substrate RNA to the lysosomal membrane through its interaction with SIDT2.

Recently, the cryoelectron microscopic structure of SIDT2 was elucidated [28, 29], suggesting that SIDT2 is not a channel or transporter. These structural insights raise the possibility that RNA may be internalized via a microautophagy-like process. Our results suggest that the function of DHX8 is independent of its enzymatic activity (Fig. 3F–I). RNautophagy mediated by both DHX8 and SIDT2 may not involve a channel/transporter that requires unwinding. Therefore, our findings in the present study are consistent with the reports that the structure of SIDT2 does not support its role as a channel or transporter.

Regarding sequence selectivity, although DHX8 was reported to preferentially bind to poly-A (10-mer) over poly-U (10-mer), poly-C (10-mer), and poly-G (10-mer) *in vitro* [21], our previous *in vitro* lysosome uptake assay showed selective incorporation of poly-G (15-mer) but not poly-A/C/U (15-mers) [7]. These observations suggest that DHX8’s *in vitro* binding preference may not directly determine the sequence selectivity of RNautophagy.

Because direct binding of recombinant DHX8 to poly-G (10-mer) was demonstrated in that study [21], the interaction observed in our pull-down assay using poly-dG (15-mer) is also likely to reflect direct binding.

Although long consecutive guanine sequences are rare in endogenous RNAs, we used poly-dG (15-mer) for pulldown screening for the following reasons. First, we previously confirmed that poly-G (15-mer) is selectively incorporated into lysosomes via RNautophagy *in vitro* [7], whereas shorter sequences have not been tested in this system, and their potential as RNautophagy substrates remains unverified. Second, we previously showed that longer sequences, such as a 15-mer, bind more strongly to the cytoplasmic domain of LAMP2C than shorter ones (e.g. 5–6 nucleotides) [7], and therefore we considered that longer oligos are more suitable for identifying a wider range of interacting proteins. How RNautophagy recognizes endogenous RNA sequences remains to be elucidated.

In WT and *Atg5* KO MEFs, knockdown of *Dhx8* reduced RNA degradation (Figs 1J, K, 2A, and B), whereas in *Sidt2* KO MEFs, RNA degradation was enhanced by the knockdown of *Dhx8* and the enhanced RNA degradation was not inhibited by CQ treatment (Fig. 2C and D). These results suggest that in *Dhx8-*knockdown *Sidt2* KO MEFs, alternative pathways, such as cytoplasmic RNA degradation, are enhanced. The molecular basis of this enhancement remains to be elucidated.

The inhibitory effect of *Eif4a*-knockdown on RNA degradation in *Atg5* or *Sidt2*-KO MEFs was more substantial than that in WT MEFs (Figs 1L and M, 2C, D, G, and H). Therefore, it is possible that the EIF4A1-dependent lysosome-independent RNA degradation pathway(s) becomes crucial when the autophagic pathway (macroautophagy or RNautophagy) is compromised. EIF4A1 may contribute to cellular RNA degradation depending on the balance among lysosomal and nonlysosomal degradation pathways.

Although the pulse-chase assay using [^3^H]-uridine effectively monitors the global degradation of endogenous RNA, it does not distinguish between RNA species. Given that rRNA constitutes the majority of total RNA, its dominant signal may mask changes in mRNA degradation. A different method is required to accurately evaluate the degradation of different RNA species, including individual mRNAs and rRNAs.

In this study, we specifically evaluated the degradation of pathogenic CAG repeat mRNAs using qPCR. The expansion of CAG repeats primarily induces neurodegeneration through cytotoxic mutant HTT proteins containing expanded polyQ regions [22]. Recent studies have suggested that eCAGr RNA can form gel-like structures (RNA foci) when lysosomal degradation is inhibited, potentially contributing to neurodegeneration [13]. In our experimental model, the knockdown of *Dhx8* reduced the degradation of eCAGr mRNA (Fig. 5E), increased eCAGr mRNA levels (Fig. 5C and D), increased the number of RNA foci (Fig. 6C and D), and elevated soluble and insoluble polyQ proteins (Fig. 7A–E). Notably, the knockdown of *Dhx8* resulted in a 20- to 40-fold increase in eCAGr mRNA levels (Fig. 5C and D), whereas the levels of soluble and insoluble polyQ only increased to ∼1.5–2-fold (Fig. 7A–E). This discrepancy may be due to an increased number of eCAGr RNA foci (Fig. 6C and D), which could subsequently suppress translation. Although further research is necessary, this study suggests that DHX8 may be involved in the pathogenesis of the disease by modulating eCAGr RNA levels and RNA foci within cells.

In addition to HTTex1, we extended our analysis to a full-length construct of ATXN3–Q79, another disease-associated protein implicated in polyQ disorders. DHX8 knockdown resulted in the accumulation of both *ATXN3*–eCAGr mRNA and its corresponding polyQ proteins, in both MEFs and N2a cells (Fig. 7F–K). These findings raise the possibility that DHX8 may regulate eCAGr RNAs across different polyQ disease conditions. In this study, we did not use disease models in which pathogenic CAG repeat RNAs are expressed under the control of their endogenous promoters. Therefore, the pathological significance of DHX8-mediated degradation remains to be clarified.

The lack of a reduction in HTTex1–CAG-145 mRNA degradation in *Sidt2* KO MEFs (Fig. 5E) might be explained by compensatory activation of alternative degradation pathways, which act on this mRNA during long-term *Sidt2* KO.

DHX8 and eCAGr RNA foci were colocalized upon inhibition of lysosomal degradation using BafA1 (Fig. 6B). It is possible that DHX8 is involved in the recruitment of eCAGr RNA or RNA foci to lysosomes during RNautophagy.

eCAGr RNA levels were increased by *Dhx8* knockdown in *Sidt2* KO MEFs (Fig. 5D) but were lower than that in Dhx8 knocked-down WT MEFs. Thus, it is possible that DHX8 regulates RNA levels through SIDT2-independent mechanisms, such as transcription. While the reduction in the rate of eCAGr RNA degradation caused by *Dhx8* knockdown was relatively modest (∼16.2%; siControl: ∼76.9%, siDhx8: ∼60.7%), the knockdown of *Dhx8* resulted in a 20- to 40-fold increase in eCAGr mRNA levels. These observations also raise the possibility that DHX8 may affect transcription, in addition to RNA degradation. The potential involvement of DHX8 in transcriptional regulation remains to be clarified, and we acknowledge this as a limitation of our current study.

In the present study, we elucidated the involvement of the RNA helicase DHX8 in RNautophagy. Our findings provide insights into the mechanisms underlying the regulation of RNA degradation, autophagic pathways, and the pathogenesis of repeat RNA-related disorders.

## Supplementary Material

gkaf801_Supplemental_Files

## Data Availability

All the source data generated for this study are available from the corresponding author (Dr Tomohiro Kabuta; kabuta@ncnp.go.jp) upon reasonable request. The mass spectrometry proteomics data have been deposited to the ProteomeXchange Consortium via the jPOST partner repository with the dataset identifier JPST003479 and ProteomeXchange identifier PXD058062.

## Notes

Present address: Department of Molecular Oncology, Institute for Advanced Medical Sciences, Nippon Medical School, Tokyo 113-8602, Japan

Present address: Łukasiewicz—PORT Polish Center for Technology Development, 54-066 Wrocław, Poland
