# Supplementary material for: DHX8 regulates degradation of RNA by RNautophagy
Source: Nucleic Acids Res. 2025 Aug 21;53(15):gkaf801. doi: 10.1093/nar/gkaf801 (PMC12370622; doi:10.1093/nar/gkaf801)
Supplement: gkaf801_Supplemental_Files [file gkaf801_supplemental_files.zip › Supplementary Figs.pdf]

SUPPLEMENTARY DATA

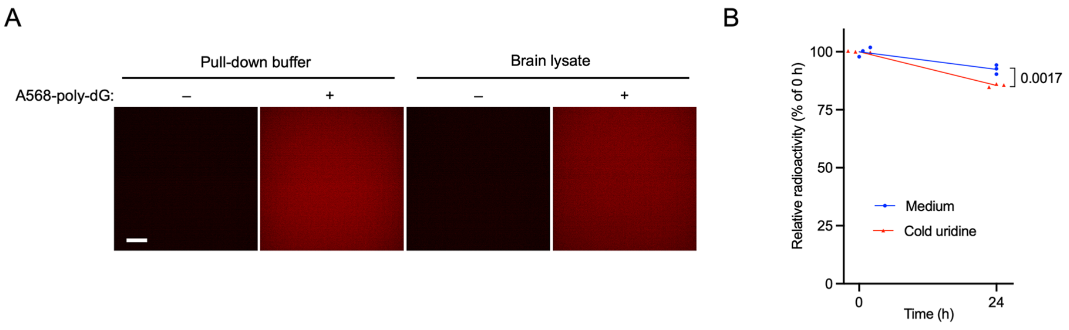

Supplementary Figure S1. Validation of assay conditions.

(**A**) We investigated whether poly-dG (15) forms foci under the pull-down conditions by using Alexa-568-labelled poly-dG (15). Alexa-568-poly-dG (15) was added at a final concentration of 1 nmol/mL to pull-down buffer or brain lysate, both prepared with the same composition as used in the pull-down assay, and imaging was performed using the SpinSR10 confocal super-resolution microscope (Olympus). Representative microscopy images are shown. No foci were observed in either solution, suggesting that protein binding to poly-dG (15) under the pull-down conditions in the present study does not depend on foci formation. Scale bar: 10 µm. (**B**) Validation of the specificity of the pulse-chase assay. WT MEFs were seeded in 24-well plates and labelled with [^{3}H]-uridine. The standard protocol, in which excess non-radioactive (cold) uridine (final 5 mM) was added to the medium during both the wash and chase steps (labelled as "Cold uridine"), with a condition in which no additional cold uridine was added in these steps (labelled as "Medium"). After a 24-hour chase, TCA-insoluble radioactivity was substantially higher in the absence of added cold uridine. This result indicates that addition of excess cold uridine effectively suppresses recycled uptake of [^{3}H]-uridine and confirms that the assay specifically monitors RNA degradation. Mean values (n = 3) are connected by lines. Individual data points are shown. P-values are from Tukey's multiple comparisons test.

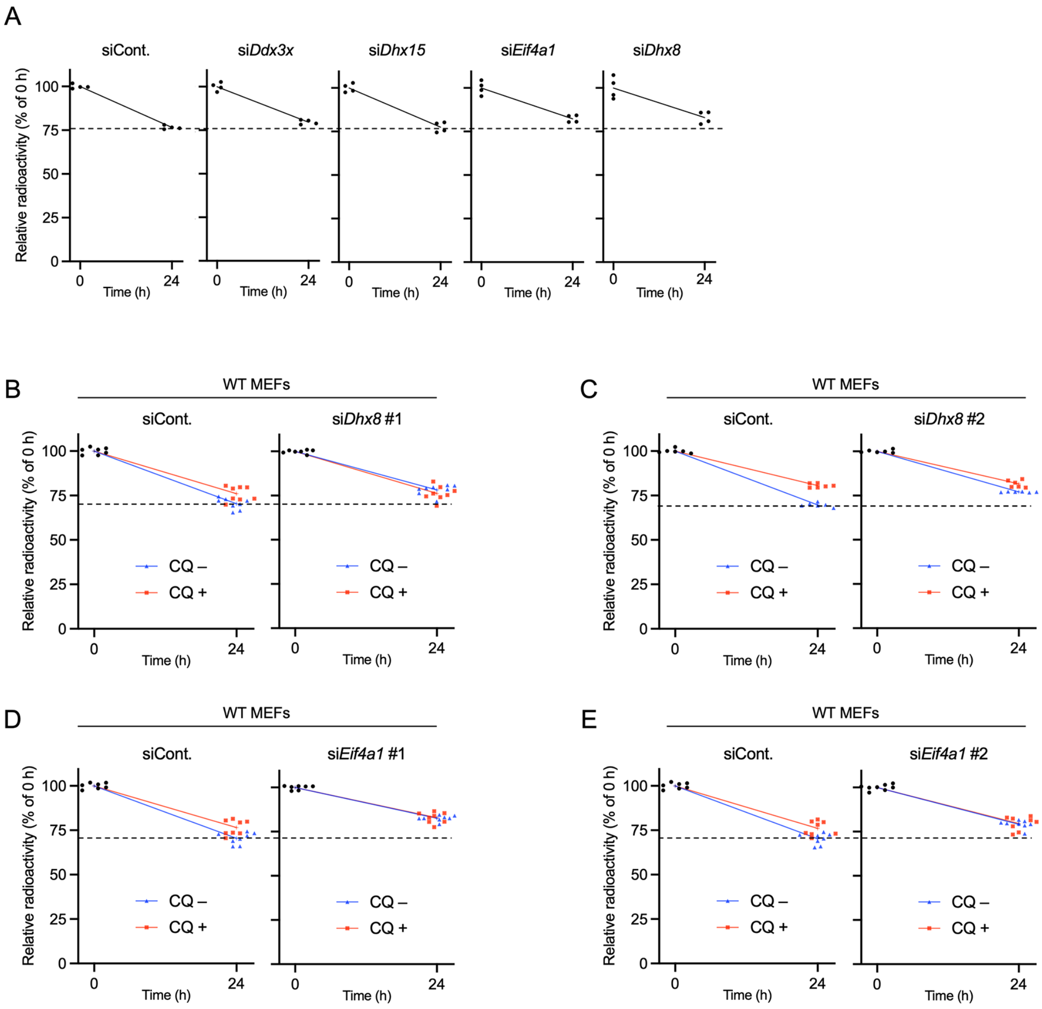

A
siCont.
siDdx3x
siDhx15
siEif4a1
siDhx8
Relative radioactivity (% of 0 h)
100
75
50
25
0
0
24
Time (h)
B
WT MEFs
siCont.
siDhx8 #1
CQ –
CQ +
C
WT MEFs
siCont.
siDhx8 #2
D
WT MEFs
siCont.
siEif4a1 #1
E
WT MEFs
siCont.
siEif4a1 #2

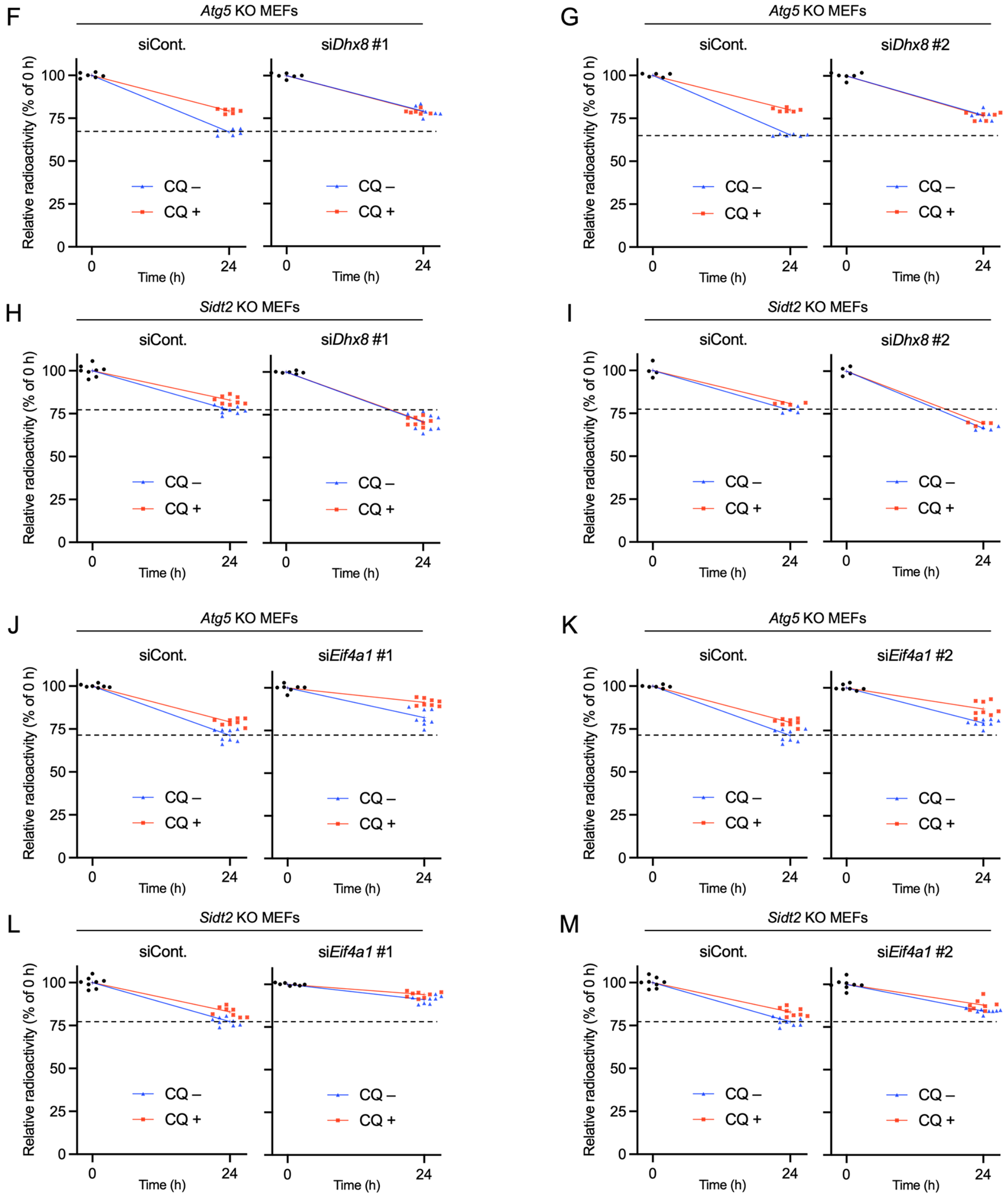
F
Atg5 KO MEFs
siCont.
siDhx8 #1
Relative radioactivity (% of 0 h)
100
75
50
25
0
0
24
Time (h)
CQ –
CQ +
G
Atg5 KO MEFs
siCont.
siDhx8 #2
H
Sidt2 KO MEFs
siCont.
siDhx8 #1
I
Sidt2 KO MEFs
siCont.
siDhx8 #2
J
Atg5 KO MEFs
siCont.
siEif4a1 #1
K
Atg5 KO MEFs
siCont.
siEif4a1 #2
L
Sidt2 KO MEFs
siCont.
siEif4a1 #1
M
Sidt2 KO MEFs
siCont.
siEif4a1 #2

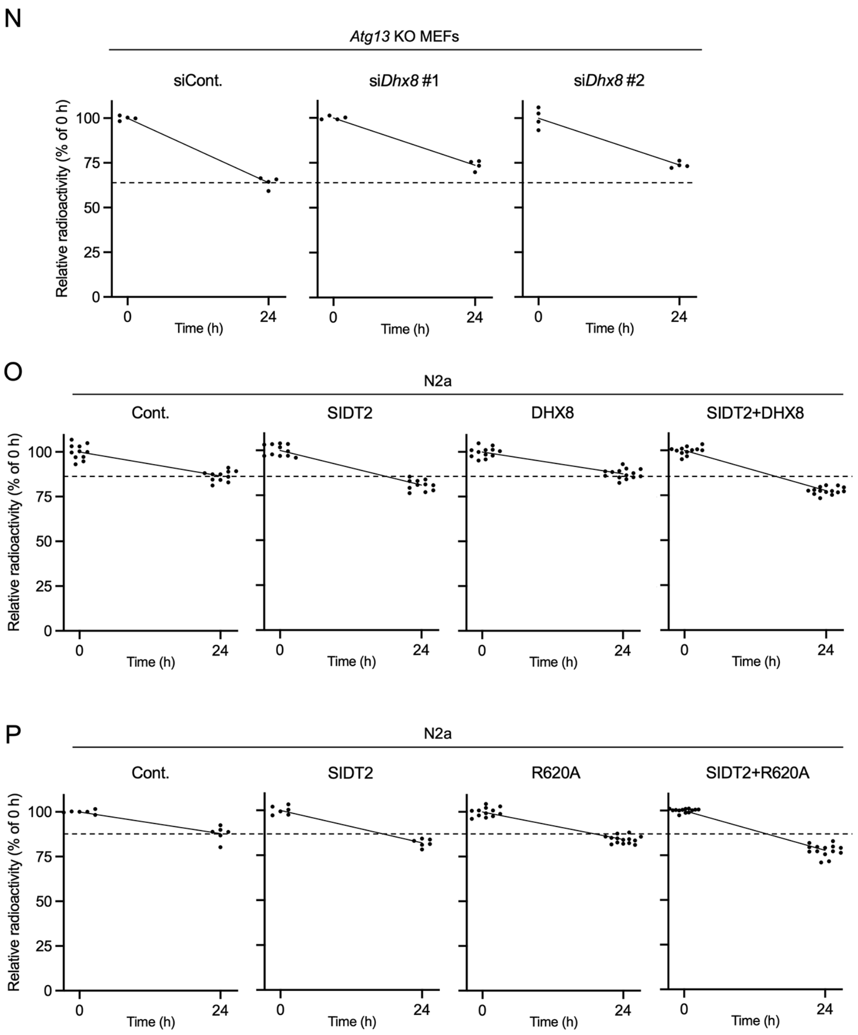

Supplementary Figure S2. Time-course data of pulse-chase assay.

(**A**–**P**) Individual time-course data points of pulse-chase assay. Panels A–E correspond to Fig. 1F, 1J, 1K, 1L, and 1M, respectively. Panels F–M correspond to Fig. 2A–2H, respectively. Panel N corresponds to Supplementary Fig. S3D. Panels O and P correspond to Fig. 3F and 3G, respectively. The Y-axis indicates relative acid-insoluble radioactivity. Dashed lines represent the mean values of control samples at 24 h in each panel.

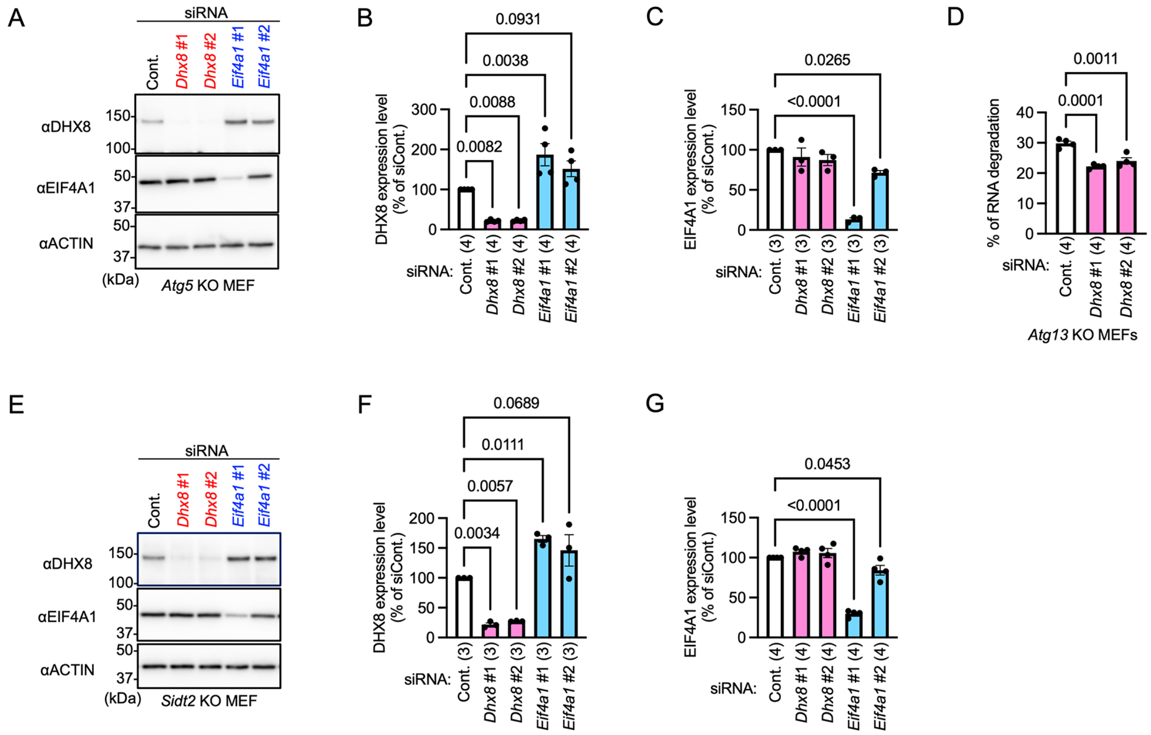

Supplementary Figure S3. RNA degradation in *Atg13* KO MEFs and knockdown efficiency of *Dhx8* and *Eif4a1*.

(**A**–**C** and **E**–**G**) Decreased levels of DHX8 and EIF4A1 proteins in *Atg5* (A–C) or *Sidt2* (E–G) KO MEFs transfected with *Dhx8* #1, *Dhx8* #2, *Eif4a1* #1, or *Eif4a1* #2 siRNA were confirmed by immunoblotting. (**D**) RNA degradation in *Atg13* KO MEFs transfected with indicated siRNAs. Data are mean ± SEM. Numbers in parentheses indicate n. P-values are from Dunnett's multiple comparisons test.

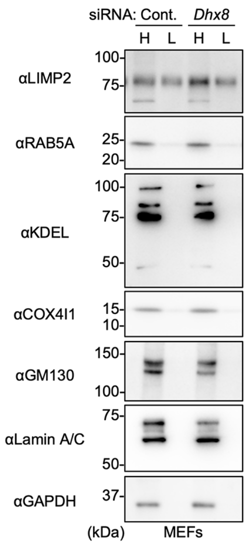

Supplementary Figure S4. Biochemical characterisation of isolated lysosomes.

Immunoblotting of lysosomes isolated from control or *Dhx8* siRNA-transfected MEFs. Homogenates (H) and lysosomal fractions (L) from these cells were subjected to immunoblotting using antibodies against the lysosomal marker LIMP2, early endosome marker RAB5A, endoplasmic reticulum marker GRP78 (anti-KDEL), mitochondrial marker COX4I1, Golgi apparatus marker GM130, nuclear marker lamin A/C, and cytosolic marker GAPDH.

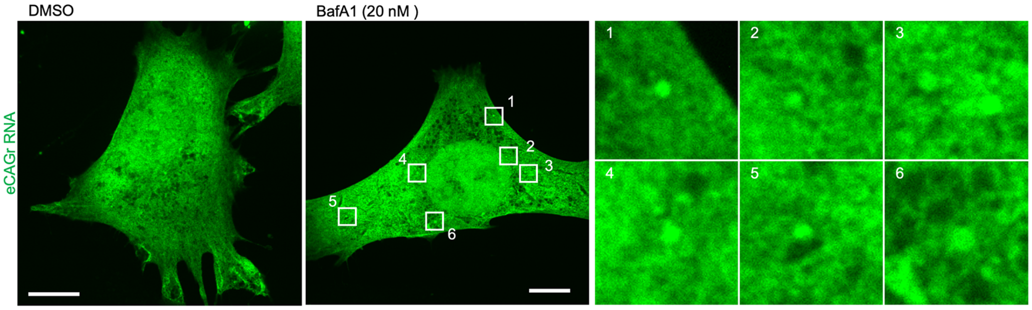

Supplementary Figure S5. Additional images of eCAGr RNA localisation under BafA1 treatment.

Zoomed views of images from Fig. 6B. Boxed regions are enlarged (right panels) to show RNA foci. Scale bar: 10 µm.

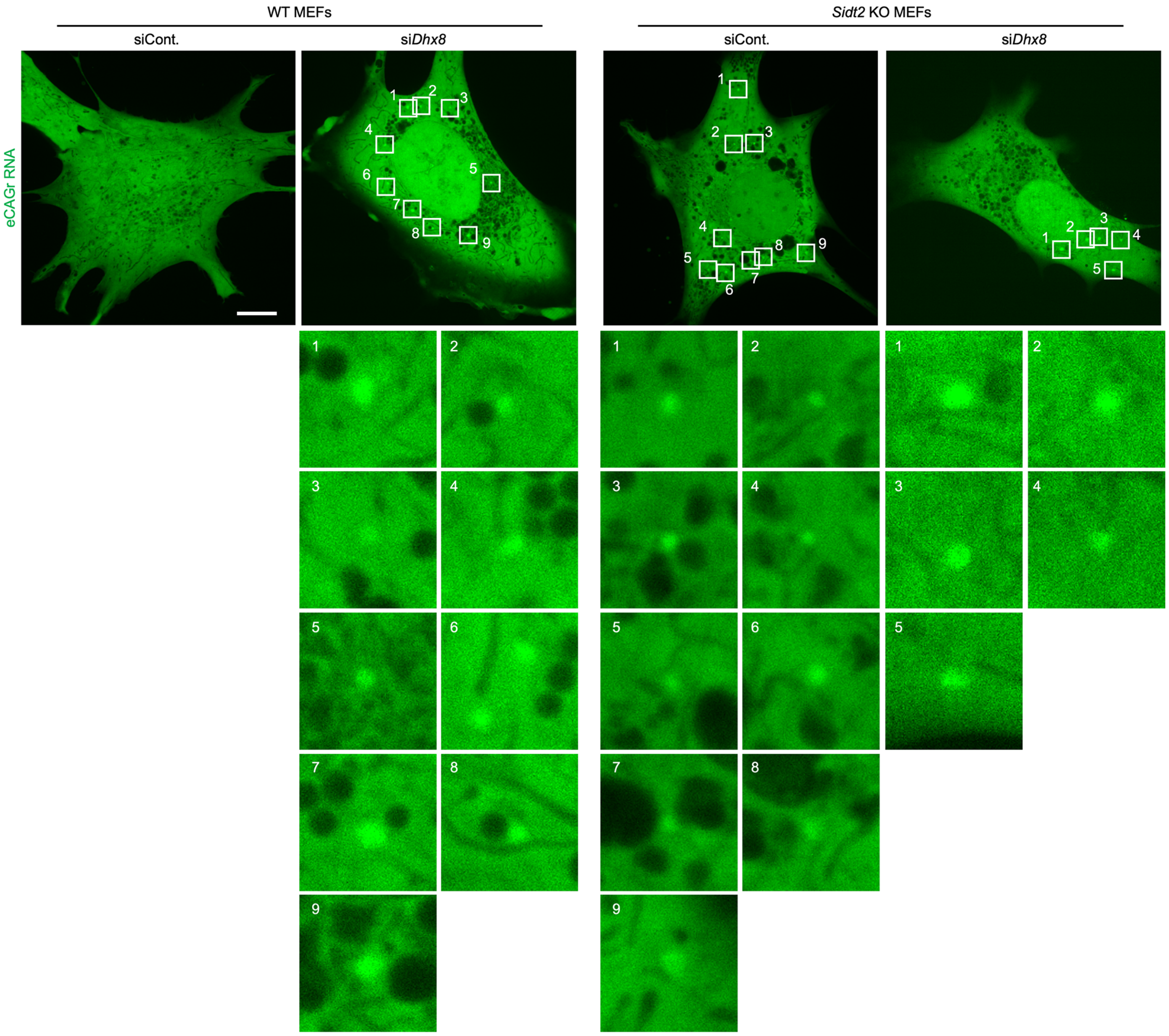

Supplementary Figure S6. Additional images of eCAGr RNA localisation upon *Dhx8* knockdown in WT and *Sidt2* KO MEFs.

Zoomed views of images from Fig. 6C. Boxed regions are enlarged (lower panels) to show RNA foci. Scale bars: 10 μm.

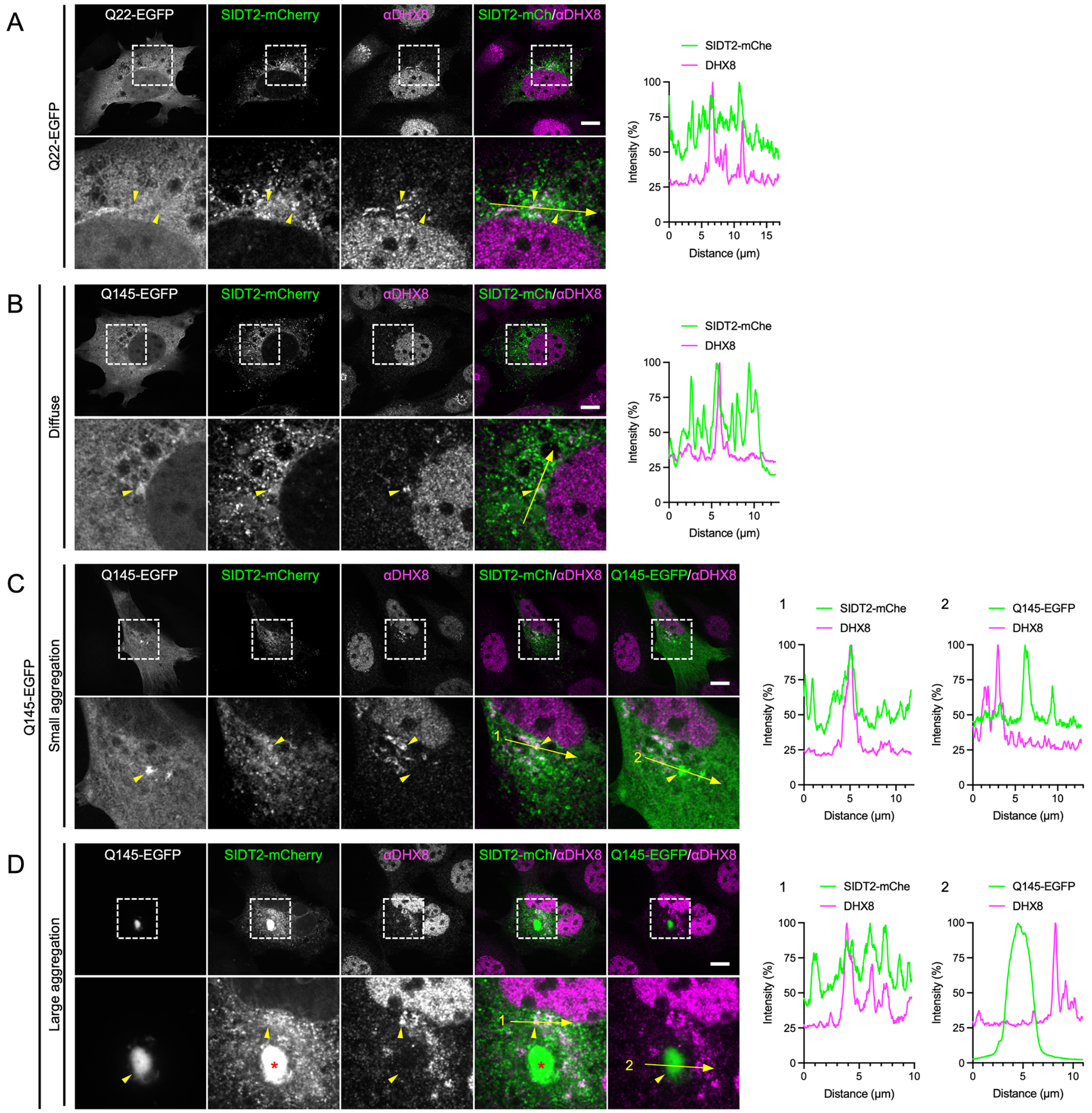

Supplementary Figure S7. Colocalisation of DHX8 and SIDT2 in the presence of expanded polyQ proteins.

(**A**–**D**) Wild-type MEFs were transfected with SIDT2-mCherry, DHX8, and HTTex1-Q22-EGFP (A) or HTTex1-Q145-EGFP (B–D). In Q22-expressing cells (A), Q22-EGFP showed a diffuse cytoplasmic distribution, and DHX8 co-localised with SIDT2-mCherry, as indicated by yellow arrowheads. In Q145-expressing cells (B–D), three distinct distribution patterns of Q145-EGFP were observed: diffuse (B), small aggregates (C), and large aggregates (D). Boxed regions are enlarged (lower panels). Line scans represent fluorescence intensity profiles along the yellow lines. Across these conditions, colocalisation of DHX8 with SIDT2-mCherry was observed. DHX8

signals did not co-localise with Q145-EGFP aggregates. Red asterisks in (D) indicate potential bleed-through of intense Q145-EGFP signal into the mCherry channel. Scale bars: 10 μm.
